# Impact of a multifaceted early mobility intervention for critically ill children — the PICU Up! trial: study protocol for a multicenter stepped-wedge cluster randomized controlled trial

**DOI:** 10.1186/s13063-023-07206-2

**Published:** 2023-03-15

**Authors:** Razvan Azamfirei, Colleen Mennie, Victor D. Dinglas, Arooj Fatima, Elizabeth Colantuoni, Ayse P. Gurses, Michele C. Balas, Dale M. Needham, Sapna R. Kudchadkar, on behalf of the PICU Up! ﻿Investigators, Mashael Alqahtani, Justin Azar, John W. Berkenbosch, Ashley R. Bjorklund, Kelly L. Corbett, Molly V. Dorfman, Andrew W. Kiragu, Matthew A. Musick, Melissa B. Porter, Charles B. Rothschild, Elizabeth E. Scarlett, Tracie C. Walker, Melvin L. Wright

**Affiliations:** 1 Department of Anesthesiology and Critical Care Medicine, Johns Hopkins University School of Medicine, Baltimore, MD USA; 2 “George Emil Palade” University of Medicine, Pharmacy, Science, and Technology, Targu Mures, Romania; 3 Division of Pulmonary and Critical Care Medicine, Johns Hopkins University School of Medicine, Baltimore, MD USA; 4 Outcomes after Critical Illness and Surgery (OACIS) Group, Johns Hopkins University, Baltimore, MD USA; 5 Department of Biostatistics, Bloomberg School of Public Health, Johns Hopkins University, Baltimore, MD USA; 6 Center for Health Care Human Factors, Armstrong Institute for Patient Safety and Quality, Johns Hopkins Medicine, Baltimore, MD USA; 7 College of Nursing, University of Nebraska Medical Center, Omaha, NE USA; 8 Department of Physical Medicine and Rehabilitation, Johns Hopkins University School of Medicine, Baltimore, MD USA; 9 Department of Pediatrics, Johns Hopkins University School of Medicine, Baltimore, MD USA

**Keywords:** Critical care, Intensive care unit, Pediatrics, Rehabilitation, Cluster randomized controlled trial

## Abstract

**Background:**

Over 50% of all critically ill children develop preventable intensive care unit-acquired morbidity. Early and progressive mobility is associated with improved outcomes in critically ill adults including shortened duration of mechanical ventilation and improved muscle strength. However, the clinical effectiveness of early and progressive mobility in the pediatric intensive care unit has never been rigorously studied. The objective of the study is to evaluate if the PICU Up! intervention, delivered in real-world conditions, decreases mechanical ventilation duration (primary outcome) and improves delirium and functional status compared to usual care in critically ill children. Additionally, the study aims to identify factors associated with reliable PICU Up! delivery.

**Methods:**

The PICU Up! trial is a stepped-wedge, cluster-randomized trial of a pragmatic, interprofessional, and multifaceted early mobility intervention (PICU Up!) conducted in 10 pediatric intensive care units (PICUs). The trial’s primary outcome is days alive free of mechanical ventilation (through day 21). Secondary outcomes include days alive and delirium- and coma-free (ADCF), days alive and coma-free (ACF), days alive, as well as functional status at the earlier of PICU discharge or day 21. Over a 2-year period, data will be collected on 1,440 PICU patients. The study includes an embedded process evaluation to identify factors associated with reliable PICU Up! delivery.

**Discussion:**

This study will examine whether a multifaceted strategy to optimize early mobility affects the duration of mechanical ventilation, delirium incidence, and functional outcomes in critically ill children. This study will provide new and important evidence on ways to optimize short and long-term outcomes for pediatric patients.

**Trial registration:**

ClinicalTrials.gov NCT04989790. Registered on August 4, 2021.

**Supplementary Information:**

The online version contains supplementary material available at 10.1186/s13063-023-07206-2.

## Background

### Background and rationale

Over 250,000 children are admitted to US pediatric intensive care units (PICUs) annually, leading to a 43% increase in the number of PICU beds since 2000 [1, 2]. While PICU mortality rates have decreased by over 50% in the last 2 decades, due to the changing spectrum of pediatric critical illness and medical advances, the decreased PICU mortality has led to a growing number of survivors experiencing both short and long-term morbidities [3–6]. Importantly, over half of all critically ill children develop preventable PICU-acquired morbidity, including iatrogenic opioid or sedative withdrawal, delirium, venous thromboembolism, pressure injury, and ICU-acquired muscle weakness, with the highest risk in mechanically ventilated patients [7–11]. These morbidities are strongly associated with poor functional recovery, which leads to decreased quality of life and increased parental stress [7, 12]. The resulting long-lasting physical, psychological, and neurocognitive impairments are known as pediatric post-intensive care syndrome (PICS-p) [3, 5, 6, 13]. The recognition of PICS-p has resulted in substantial research in adult ICU populations and is recognized by the NIH/NHLBI and major academic societies as a high research priority [14–17]. Thus, the preventable morbidities experienced by a growing number of PICU survivors are an important public health issue urgently in need of evidence-based strategies.

Early and progressive mobility is associated with improved outcomes in critically ill adults including shortened duration of mechanical ventilation and improved muscle strength [18–23]. However, the clinical effectiveness of early and progressive mobility in the PICU has never been rigorously studied. Unlike adults, critically ill children are admitted to the PICU during a crucial period of physical and neurocognitive development. Less than 40% of PICU survivors with normal baseline function recover to their baseline by 3 months after PICU discharge [7]. Moreover, our multicenter point prevalence study of physical rehabilitation in 82 US PICUs found that 68% of all admissions ≥ 3 days are patients ≤2 years old [24]. Thus, there is an urgent need to attend to both rehabilitation and habilitation, the acquisition of new physical and cognitive skills, in the PICU.

Despite the known harms of bed rest, a PICU culture of immobility is perpetuated by oversedation, physical restraints, and poor sleep hygiene due to a perceived need to optimize safety and comfort [25–27]. These factors all increase the risk of prolonged mechanical ventilation [28]. Single-component approaches to decrease the duration of mechanical ventilation in the PICU have not consistently shown effectiveness [29–32]. These issues have been mitigated in adult ICUs with early rehabilitation as part of the interprofessional ABCDEF bundle [33, 34]. The ABCDEF bundle is associated with lower mortality, less delirium, and more ventilator-free days in critically ill adults [33–35]. However, the heterogeneity of the PICU patient population limits the direct translation of this approach and has never been rigorously studied.

### Objectives

The objective of the study is to evaluate if the PICU Up! intervention, delivered in real-world conditions, decreases mechanical ventilation duration (primary outcome) and improves delirium and functional status compared to usual care in critically ill children. A secondary objective is to conduct a multi-stakeholder, mixed-methods process evaluation to identify key contextual factors associated with the delivery of PICU Up!.

### Trial design

This study is a mixed-methods, stepped-wedge, cluster randomized superiority trial of a pragmatic, interprofessional and multifaceted early mobility intervention (PICU Up!). The PICU Up! trial will include pediatric intensive care units (PICUs) from 10 discrete hospitals in the United States, with each PICU acting as one cluster. In this traditional stepped-wedge design, all clusters begin in the control group and then transition to the intervention group at sequential and randomly assigned periods, facilitating the delivery of the desired intervention to all clusters. Thus, randomization will occur at the level of the PICU unit. All participating PICUs will simultaneously start the baseline control period and data collection and plan their PICU Up! implementation process. The study includes an additional embedded process evaluation to identify factors associated with reliable PICU Up! adoption and performance. This study protocol is reported in accordance with the SPIRIT (Standard Protocol Items: Recommendations for Interventional Trials) guidelines [36, 37].

## Methods and analysis

### Study setting

The PICU Up! trial will include 10 PICUs in 10 states within the US representing a diverse mix of public, private, and federally funded teaching and nonteaching hospitals, of various sizes and patient populations; all included PICUs do not have an existing PICU mobility protocol and will not implement a mobility protocol until their randomized time of implementation. Participating sites were identified through informal surveys of pediatric critical care investigators and investigators participating in the Pediatric Acute Lung Injury and Sepsis Investigators (PALISI) Network. Historical PICU admission numbers were evaluated to ensure that the overall patient enrollment target is feasible.

### Eligibility criteria

**Fig. 1:**
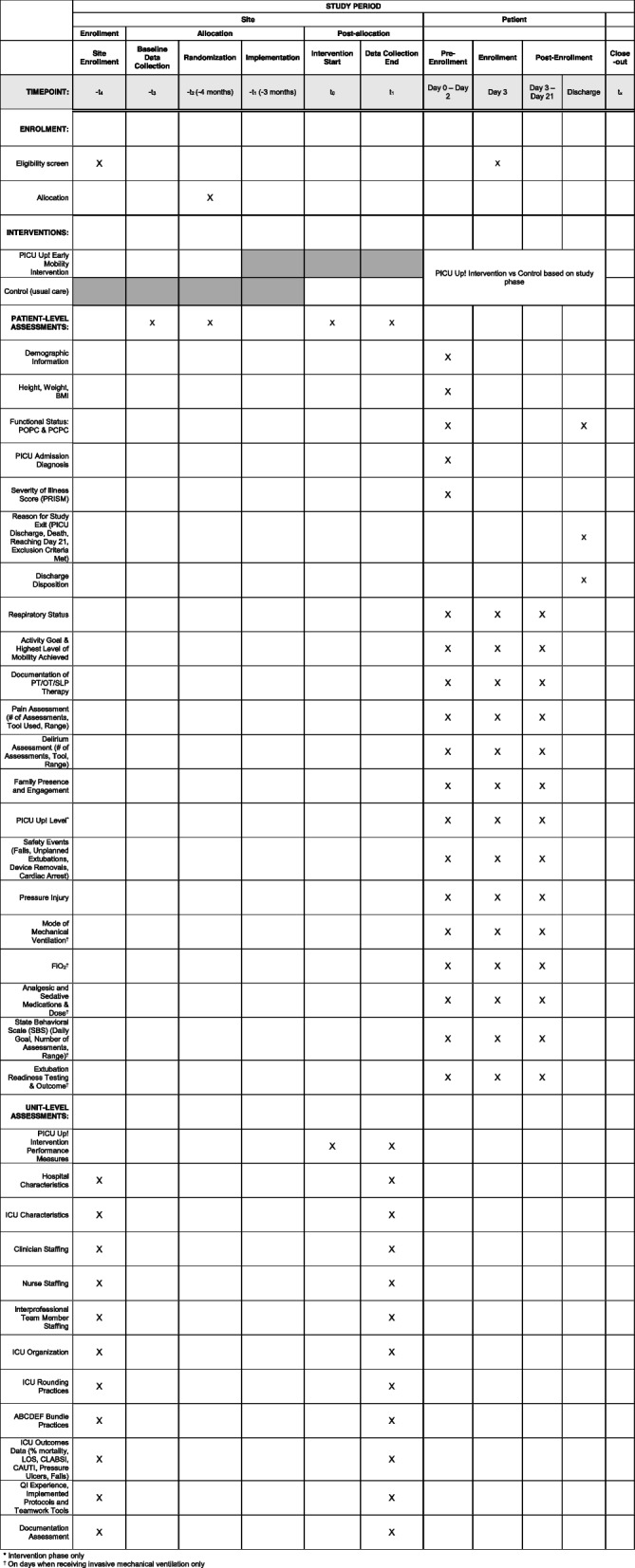
Schedule of Enrolment, Interventions, and Assessments. * Intervention phase only. ^†^ On days when receiving invasive mechanical ventilation only

All PICU patients, regardless of their length of stay, are screened for the PICU Up! unit-based intervention. Patients receiving invasive mechanical ventilation ≥ 48 h on day 3 of the PICU admission will be included in the study. Patients with active or anticipated withdrawal of life support within 48h, with an open chest or abdomen, or receiving extracorporeal membrane oxygenation (ECMO) will be excluded from the study (Fig. 1).

### Intervention

**Fig. 2:**
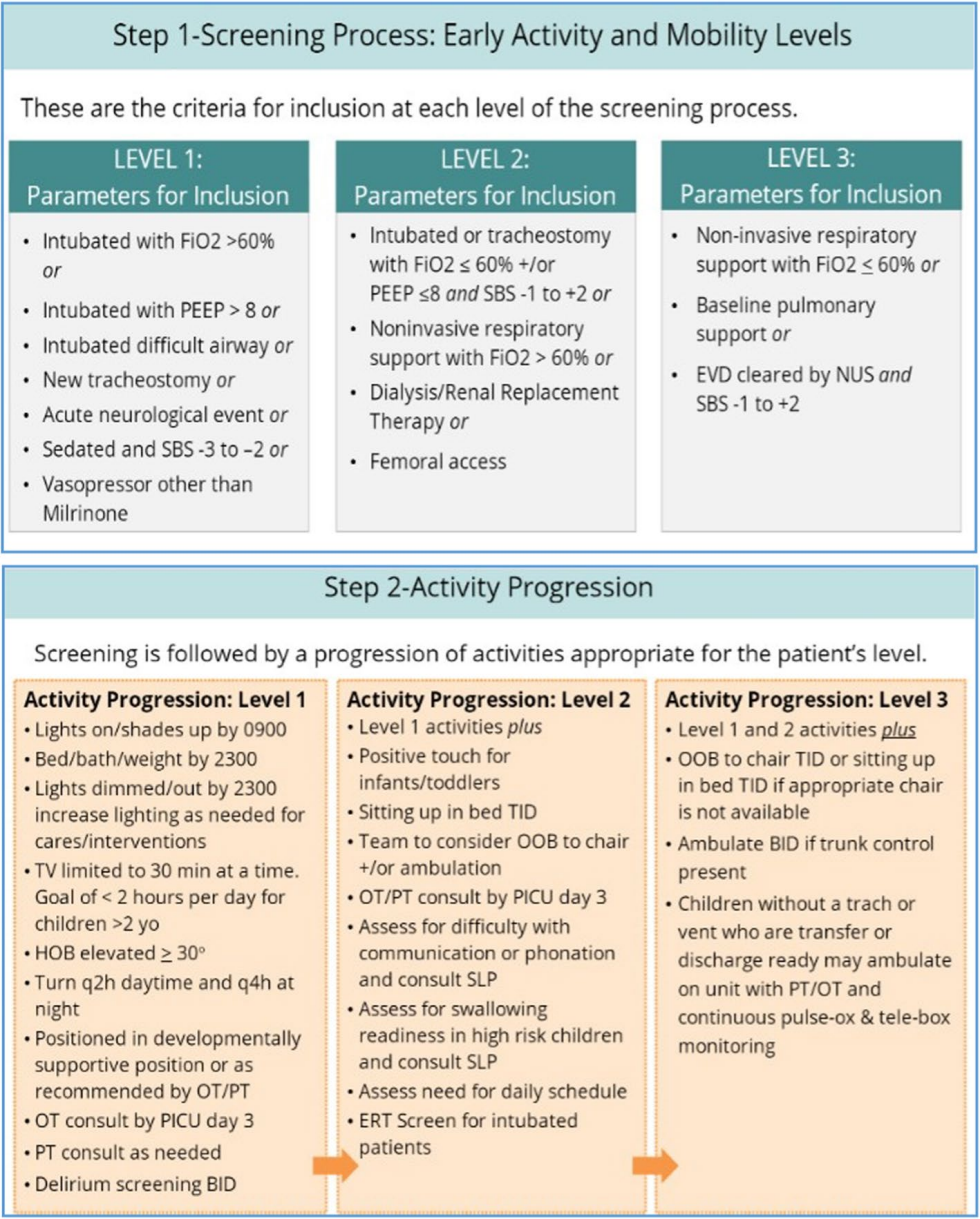
PICU Up! levels and corresponding activities

PICU Up! is a multifaceted, interprofessional, and systematic tiered pathway that is integrated into routine PICU practice to safely optimize early and progressive mobility. Detailed methodology for the development of the PICU Up! intervention has been published [38]. PICU Up! incorporates the screening process for determining a patient’s appropriate activity level into the daily rounding workflow for all PICU patients, with a tiered activity plan (Fig. 2) based on clinical parameters to individualize goals based on each child’s unique needs. Included are criteria for pausing activity and for reassessing the patient before continuing the activity [38]. While the PICU Up! level is based on objective criteria, the interprofessional team collectively determines the daily activity goal(s) through shared decision-making, which is documented in the medical record on morning rounds. The intervention includes a rounding template which ensures daily discussion of the PICU Up! level and elements: (1) analgesia; (2) protocolized extubation readiness testing; (3) sedation level and goal; (4) delirium screening; (5) mobility goal including PT/OT consultation by day 3; (6) family engagement in mobility; and (7) sleep promotion. As a pragmatic trial, all other aspects of PICU care will be conducted per routine practice.

Prior to and during the baseline data collection phase, the clinical sites will develop a PICU Up! implementation strategy with guidance and resources from the Clinical Coordinating Center (CCC). The implementation planning will focus on local barriers and facilitators to early and progressive mobility for critically ill patients as well as goal-directed sedation, delirium prevention, extubation readiness assessments, sleep promotion, and family engagement. The PICU Up! Implementation Bundle includes discipline-specific educational resources (webinars, templates, educational presentations) and a unit-based electronic learning module to orient all PICU staff to PICU Up!.

As the intervention is administered by the clinical team to critically ill children, there no concerns related to patient adherence. Clinical team adherence to the core elements of the PICU Up! intervention will be monitored at both the patient and the unit level. Each site will receive periodic reports summarizing the PICU Up! compliance metrics. Individualized support will be provided by the CCC to sites to address adherence challenges.

### Timelines

**Fig. 3:**
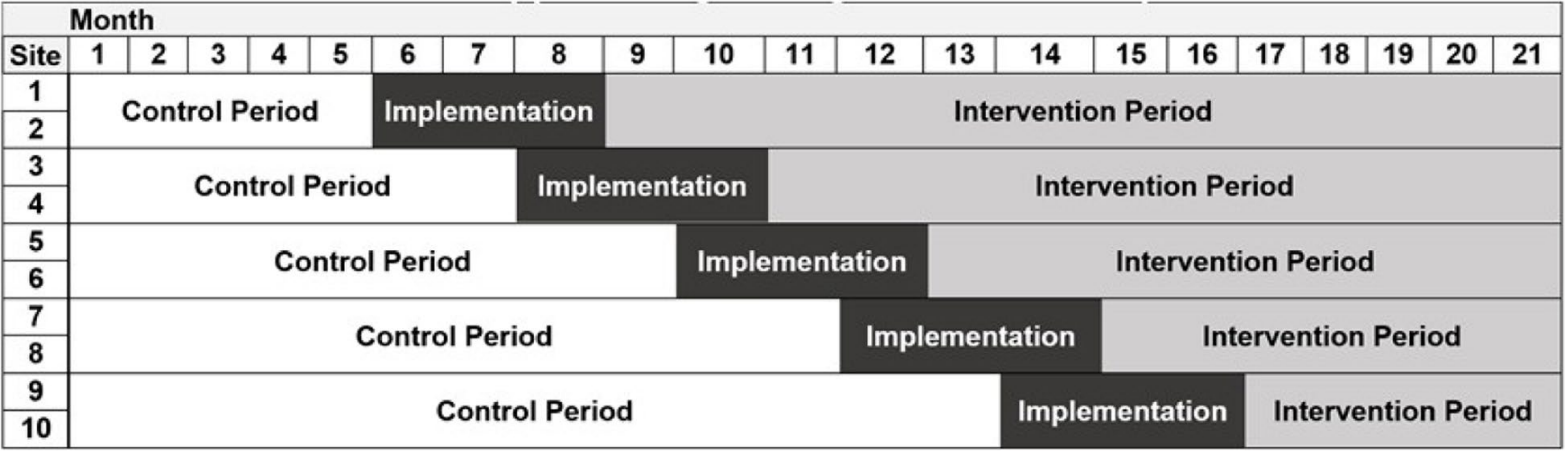
Stepped-wedge design for the PICU Up! trial

After IRB approval and setup for data collection, all participating PICUs will simultaneously start the control period with data collection and continue implementation planning (Fig. 3). The order in which the PICUs will move into the intervention period will be determined through randomization. Each site will be notified of its transition date 4 months in advance. PICU Up! will be implemented over the following 3 months including PICU staff training. All PICUs will remain in the study for the entire study duration (i.e., 21 months), with each pair of PICUs being exposed to the intervention for different durations based on the randomization schedule.

Identical, standardized data collection will occur during both the control and intervention phases. Figure 1 provides a timeline for enrolment, assessments, and interventions.

### Outcome measures

#### Primary outcomes

Our primary patient-level composite outcome is the number of days alive and ventilator-free (AVF) within the first 21 days after intubation or after PICU admission for patients intubated at an outside hospital. Days AVF within 21 days are defined as days when the patient was alive, and an endotracheal tube was absent for 24 continuous hours during a calendar day, ranging from 0 (any patient who experiences death prior to extubation or those intubated for more than 21 days) to 20 (patient is extubated the day following intubation and is alive by 21 days). If the patient was successfully extubated and discharged before 21 days, the days after discharge are ventilator-free. For patients discharged to a long-term ventilation rehabilitation facility, if such discharge happens before day 21, the patient will be considered to be mechanically ventilated for the remainder of the days to day 21. Days AVF was selected over the rank-based composite ventilator-free days given that expected mortality rates are relatively small and days AVF considers any day off the ventilator as important to the patient, family, and provider regardless of patient survival [4, 39–41].

#### Secondary outcomes

Secondary outcomes will include days alive and delirium- and coma-free (ADCF), days alive and coma-free (ACF), and days alive, as well as functional status at the earlier of PICU discharge or day 21, measured through Pediatric Cerebral Performance Category and Pediatric Overall Performance Category scores. If the patient was discharged from the PICU alive, all days following discharge will be considered days alive, delirium- and coma-free (Table 1).

**Table 1:** Definition of outcome measures

**Primary outcome**	
Days alive and ventilator-free (AVF)	Days when the patient was alive, and an endotracheal tube was absent for 24 continuous hours during a calendar day^a,b^ through 21 days
**Secondary outcomes**	
Days alive and delirium- and coma-free (ADCF)	Days when the patient was alive, without delirium, and coma-free for 24 continuous hours during a calendar day^b,c,d^ through 21 days
Days alive and coma-free (ACF)	Days when the patient was alive, and coma-free for 24 continuous hours during a calendar day^b,d^ through 21 days
Days alive	Days when the patient was alive for 24 h during a calendar day^e^ through 21 days
Functional status	Pediatric Cerebral Performance Category and Pediatric Overall Performance Category scores assessed at the earlier of PICU discharge or day 21
**Exploratory outcomes**	
PICU length of stay	Number of days on which the patient was physically present in the PICU rounded to the nearest higher day through 21 days
PICU mortality	Number of patients deceased or withdrawn from the study for limitation of care orders or brain death evaluation/total enrolled patients
PICU and hospital discharge destination	Number of patients discharged to home, inpatient floor, inpatient, rehabilitation, other hospitals/number of discharged patients
New pressure injuries	Rate of new pressure injuries/eligible patient days through 21 days
Opioid exposure	Opioid dose (mg/kg/day) measured in morphine milligram equivalents (MME)^f^
Benzodiazepines exposure	Benzodiazepine dose (mg/kg/day) measured in midazolam milligram equivalents^g^

^a^ For patients with a tracheostomy, a ventilator-free day will be defined as a day when the patient is not receiving invasive mechanical ventilation for 24 continuous hours during a calendar day. Specifically, patients with a tracheostomy whose breaths are unassisted (i.e., trach collar) will be considered ventilator-free; patients receiving assisted breaths (i.e., CPAP) will not be considered ventilator-free

^b^ Days following PICU discharge to 21 days will be considered days alive, ventilator-, delirium- and coma-free, with the following exceptions: days after PICU discharge will not be considered days alive for deceased patients, days after PICU discharge will not be considered days ventilator-free if the patient remains intubated at discharge

^c^ Delirium-free days are days when the patient is at risk of delirium (i.e., coma-free) and delirium was not present

^d^ Coma-free days are days when all documented sedation scores are above the following thresholds: SBS ≥ − 1; RASS ≥﻿ −3; COMFORT-B ≥﻿ 10

^e^ For patients who were withdrawn from the study due to limitation of care orders or brain death evaluations, they will be considered deceased on the day death is pronounced

^f^ The following conversion will be used: fentanyl: 10 mcg = 1 MME; hydromorphone: 0.25 mg = 1 MME; morphine 1 mg = 1 MME [42]

^g^ The following conversion will be used: lorazepam: 0.5 mg = 1 midazolam milligram equivalents; midazolam: 1 mg = 1 midazolam milligram equivalents [43]

#### Exploratory outcomes

Exploratory outcomes will include PICU length of stay, PICU mortality, PICU and hospital discharge destination, presence of new pressure injuries, as well as exposure to opioids and benzodiazepines (Table 1).

#### Process evaluation

A mixed-methods process evaluation will be performed using the UK’s Medical Research Council’s framework for evaluating complex interventions and guided by the Systems Engineering in Patient Safety 2.0 Framework (SEIPS 2.0; Preliminary Data) [44].

We will summarize the outcome of PICU Up! performance at the PICU level for each month of the intervention period. Specifically, we will calculate a measure of complete, proportional, and individual element performance for each PICU (Table 2). The monthly complete and proportional performance will respectively be defined as the proportion of patient days (for eligible patients) in which every eligible element of PICU Up! was performed and the proportion of eligible elements received divided by the number of eligible elements [34]. Individual performance will be defined as the proportion of patient days (for eligible patients) where the individual element was received divided by the number of days where the individual element was eligible.

**Table 2:** Definition of PICU Up! performance measures [34]

Element	Days eligible	Performance
**A**	All days	≥ 6 pain assessments/24 h using a validated pediatric pain scoring scale
**B**	Days when receiving invasive mechanical ventilation	Screened for extubation readiness trial once daily if receiving invasive mechanical ventilation
**C**	Days when receiving sedation and/or invasive mechanical ventilation	≥ 6 sedation assessments/24 h using a validated sedation scale and sedation goal documentation if receiving sedation and/or invasive mechanical ventilation
**D**	Days when the patient is at risk for delirium	≥ 2 delirium screening assessments using a validated pediatric delirium screening tool
**E**	All days	Documentation of mobility goal AND highest level of mobility achieved each day
**F**	All days	A family member was educated on PICU Up! and/or participated in rounds/conferences/mobility

To examine implementation factors, we will analyze copies of all PICU Up!-related documentation forms and policies at study initiation and at the end of the study’s data collection phase to identify if sites adapted any of the early mobility screening process criteria or activity progression steps, utilized any of the provided EHR templates, or revised pain, sedation, delirium, mechanical ventilation, mobility, or sleep protocols. Additionally, we will analyze contextual factors by administering a validated organizational and resource availability survey that collects hospital and ICU organizational characteristics, utilization rates, staffing patterns/ratios, and rounding and PICU Up! practices at the beginning of the study and at the end of the study’s data collection period [24, 34, 45].

### Sample size and power calculation

Site-specific admissions data previously published trial data, and data from the PICU Up! pilot multicenter trial were used to conservatively estimate that an average of 8 patients/cluster/month will be eligible for inclusion for data collection [24]. In the proposed stepped-wedge design, with two hospitals switching at each of the five steps, a total of 180 months of observation (90 months control/90 months intervention) will be available for analysis. Therefore, 720 patients are expected to be included in the baseline period and 720 in the intervention (post-implementation) period. The power calculation accounted for the clustered nature of the study design and the confounding effect of time using preliminary data obtained from our multicenter pilot trial [46]. Specifically, 1000 hypothetical stepped-wedge trials were simulated assuming patients within hospitals would be correlated with an intra-class correlation (ICC) of 0.01 and allowed the days AVF to day 21 to decline linearly over time (month 1’s mean (variance) of 13.2 (26.4) days AVF to day 21, with a monthly decline of 0.1 days) with a fixed effect of the intervention that is constant over time. Given the design, the anticipated sample size, type I error of 5%, and power of 80%, a ≥ 1.8-day improvement in mean days AVF to day 21 will be detectable between the intervention and control period, which is a clinically meaningful reduction in the duration of mechanical ventilation [29–31].

### Allocation

The order in which the PICUs will move into the intervention period will be determined through randomization. Two PICUs will cross over to the intervention during each of the five steps. All possible permutations will be considered for the order of transition from control to intervention. Given that some permutations will lead to an imbalance in patients included in the control and intervention phases, the imbalance for each permutation will be estimated based on anticipated PICU-specific eligibility expectations, and permutations with excessive imbalance will be excluded. The permutation selected for implementation will then be chosen at random from permutations with adequate balance. The set of unique permutations, imbalance scoring, and assignment will be performed using the R statistical package.

### Blinding

Given the nature of the intervention, it is not possible to blind this study to patients, clinicians, or research staff. However, the primary outcome is objective and quantitative and unlikely to be affected by the care team’s perceptions or behaviors.

### Data collection and management

Identical, standardized data collection will occur during both the control and intervention phases. Data collection includes patient demographic, clinical, and rehabilitation data from the Epic electronic health record (EHR) from PICU admission through the earlier of day 21 or PICU discharge. The Data Coordinating Center (DCC) and CCC will provide oversight for all relevant study-related activities. The composition of the DCC and CCC are specified in Additional file 1. Data will be captured, managed, and exported using a secure, auditable, and HIPAA-compliant database (REDCap) with unique de-identified research participant identifiers [47]. The REDCap database will include automated data validation, range checks, branching logic, and designating fields as required. Data quality will be ensured through protocolized data collection, staff training and re-training on protocols, and auditing, independent of investigators and the sponsor. Auditing by the DCC includes initial and ongoing quality assurance procedures, periodic data cleaning, regular data queries, and PICU Up! performance feedback to sites [48].

The clinical trial will collect data on potential adverse events prospectively; this data will be reviewed daily, and reports will be made to the Data Safety Monitoring Board (DSMB), Institutional Review Board (IRB), and sponsor as required.

### Statistical methods

#### Patient-level outcomes

Descriptive statistics for continuous variables will be presented as the mean ± standard deviation and quantiles and categorical variables will be expressed as percentages. The effect of the PICU Up! intervention on the clinical outcomes will be estimated using mixed-effects models that include indicators for both the implementation (3-month duration) and the intervention vs. control periods and incorporate features of the stepped-wedge design by including a random intercept defined for each site to account for clustering of outcomes within sites and a fixed effect of time (month, modeled as a natural cubic spline) to account for temporal trends in the outcomes over the period of the study [49]. Linear mixed-effects models will be used for the primary outcome where the coefficient for the indicator of intervention vs. control period directly estimates the mean difference in days AVF by day 21 comparing the intervention and control period. To account for potential violations of model assumptions, robust variance estimates will be used. Similar linear mixed-effects models will be used for the secondary outcomes. We expect that the distribution of the primary outcome and the secondary outcomes will be skewed; however, given the large sample size, the linear models should be robust for estimating the mean difference in these outcomes. Poisson log-linear mixed-effects models with the same random and fixed effects will be used as a sensitivity analysis for the linear mixed-effects models proposed above. Logistic mixed-effects models will be used for binary patient-level outcomes. For the ordinal outcomes, Pediatric Cerebral Performance Category and Pediatric Overall Performance Category, if sufficient data is present in each ordered category or if we collapse the 6 ordered categories into at least 3 categories, we will utilize multinomial models to evaluate the effect of the intervention. If the data suggest collapsing the ordered categories into only two categories, then binomial regression models will be used.

The primary analysis for all outcomes will be unadjusted, that is, we will assume that patients enrolled in the study over time will be similar with respect to characteristics associated with the outcomes (e.g., age and severity of illness). Secondary analyses will include exploratory analyses to evaluate this assumption and the models (described above) will be extended to include an adjustment for key patient characteristics (e.g., age, admission diagnosis category, measures of baseline function such as PCPC, POPC, FSS). Analyses to detect significant differences in the intervention effect among sex/gender will be conducted. Additional secondary analyses will fit the models described above, including adjustment for the patient-specific measure of PICU Up! performance by separately including the measure of proportional performance (ranging from 0 to 1) and the binary indicator of a complete performance.

Statistical significance will be set at *p*-value < 0.05 using a two-sided hypothesis test for the primary outcome analysis; all secondary analyses will be considered exploratory with two-sided 95% confidence intervals reported. We will conduct missing data assessment/estimation, and if necessary, employ methods for imputation of missing data using the MICE package [50].

#### Process evaluation

Online survey data will be summarized using descriptive statistics. The mean and standard deviation will be reported for continuous measures and frequency and percentages for categorical variables. Trends in performance within a PICU over time will be explored using spaghetti plots with locally weighted regression smoothers to explore the general trends over time across all PICUs. Mixed-effects Poisson models will be used to model the trends in performance over time via restricted cubic splines with a minimum of a random intercept defined at the PICU level. Subsequently, the models will include the hospital and PICU-level covariates, such as structural characteristics, staffing patterns, organizational traits, rounding practices, and the use of daily teamwork tools. The inclusion of such covariates will allow for the determination of whether variation in performance across the PICUs is partially explained by these PICU-level factors.

#### Interim analyses

Interim analyses will be performed and presented to the DSMB every 12 months from the beginning of data collection. The interim analyses will focus on patient accrual and safety; data on trial operations, gender and minority inclusion, and intervention effects will also be reviewed. The DSMB may recommend stopping the trial if (1) the intervention is associated with increased safety events, (2) patient accrual is well below acceptable goals and the ability of the study to achieve its goals is seriously compromised, or (3) evidence external to the study renders it unethical to continue the study. Given the minimal anticipated risk of the PICU Up intervention, no a priori stopping rules were specified for early stopping for safety or futility of the intervention.

### Ethics and dissemination

Johns Hopkins Medicine is serving as the single IRB for the study and all sites must obtain either local IRB acknowledgment or IRB approval if unable to participate in single IRB oversight. Ethical approval was obtained from the Johns Hopkins Medicine IRB (IRB00297110). All eligible subjects at participating sites will have data collected under a waiver of informed consent provided by the Johns Hopkins Medicine IRB. The trial will not collect any biological specimens for the participant and given the nature of the study and the approved waiver of consent there will be no individual consenting process. The study uses the waiver of consent process based on the following factors: (1) the intervention is targeted to the PICU care environment and interprofessional shared decision-making and does not deviate from accepted clinical practice; (2) the intervention poses no more than minimal risk; (3) the intervention involves no procedures for which written consent is normally required outside of the research context; (4) this research could not practicably be carried out without a waiver of consent, the waiver will not adversely affect the rights and welfare of the subjects; and (5) obtaining informed consent would threaten the scientific validity of the study, which depends on capturing all eligible patients during the enrollment period. Whenever appropriate, patients and their families will be provided with additional pertinent information after participation and can decline any of the PICU Up! interventions. Given the minimal risk nature of the study and the fact that the intervention does not deviate from accepted clinical practice, there are no provisions for any ancillary or post-trial care nor is any compensation available for any potential harm resulting from trial participation.

The Clinical Coordinating Center will maintain a study website that can only be accessed by approved study team members from each participating site. The website will contain the most current version of the protocol, amendments to the protocol, and educational and study-related resources. These study materials will also be distributed via email to the site principal investigator and research coordinators as they are available or updated.

Data generated in this grant will be presented in a timely fashion at national and international meetings and in publications. All final peer-reviewed manuscripts that arise from this proposal will be submitted to the digital archive PubMed Central. Authorship eligibility will be determined using ICMJE Guidelines. The investigators plan to share deidentified data from this research with other researchers who have an interest in improving outcomes in critically ill children through early and progressive mobility interventions after review of a written proposal and establishment of institutional data use agreements.

## Discussion

The PICU Up! trial is the largest pediatric trial examining the effectiveness of a pragmatic, interprofessional, and multifaceted early mobility intervention in improving ICU outcomes. Bundled, interdisciplinary interventions have been shown to improve ICU outcomes in adult populations, leading to the widespread adoption of ICU liberation bundles [34, 35, 45]. However, such interventions have not been widely implemented or examined rigorously in pediatric populations. PICU Up! is a unit-based intervention that is directed at optimizing interprofessional collaboration to integrate goal-directed sedation, extubation readiness assessments, sleep promotion, delirium prevention, early mobility, and family engagement into the daily PICU workflow [38]. As a unit-based intervention, each child in the PICU is screened for PICU Up! eligibility regardless of their acuity of illness or mechanical ventilation status, and receives interventions based on their physiologic status and shared decision-making by the interprofessional team.

This study offers a potential benefit to patients as it is aimed at finding methods to improve the outcomes of pediatric survivors of critical illness, many of whom experience persistent physical, psychological, and neurocognitive impairments. This study will contribute to understanding whether a multifaceted strategy to optimize early mobility affects the duration of mechanical ventilation, delirium incidence, and functional outcomes. Consequently, this study will generate important new knowledge regarding ways to optimize short and long-term outcomes for critically ill children.

### Trial status

Patient enrollment began on August 1, 2022, and will conclude on June 1, 2024. The current version of the protocol, version 2.0, was approved on February 20th, 2023. Version 2.0 is the first revision of the protocol.

## Supplementary Information

**Additional file 1.** Trial Protocol.

## Data Availability

The principal investigator, members of the CCC and DCC, as well as the study statistician will have access to the final dataset. The investigators plan to share deidentified data from this research with other researchers who have an interest in improving outcomes in critically ill children after review of a written proposal and establishment of institutional data use agreements.

## Abbreviations

ACF: Alive and coma-free
ADCF: Alive and delirium- and coma-free
AVF: Alive and ventilator-free
BMI: Body Mass Index
CAUTI: Catheter-associated Urinary Tract Infections
CCC: Clinical Coordinating Center
CLABSI: Central Line-associated Bloodstream Infections
DCC: Data Coordinating Center
DSMB: Data and Safety Monitoring Board
ECMO: Extracorporeal Membrane Xxygenation
FiO_2_: Fraction of inspired Oxygen
ICU: Intensive Care Unit
IRB: Institutional Review Board
LOS: Length of Stay
OT: Occupational Therapy
PCPC: Pediatric Cerebral Performance Category
PICS-p: Pediatric Post-Intensive Care Syndrome
PICU: Pediatric Intensive Care Unit
POPC: Pediatric Overall Performance Category
PT: Physical Therapy
PRISM: Pediatric Risk of Mortality
QI: Quality Improvement
SBS: State Behavioral Scale
SLP: Speech-Language Pathology
