# Supplementary material for: Impact of a multifaceted early mobility intervention for critically ill children — the PICU Up! trial: study protocol for a multicenter stepped-wedge cluster randomized controlled trial
Source: Trials. 2023 Mar 15;24:191. doi: 10.1186/s13063-023-07206-2 (PMC10015670; doi:10.1186/s13063-023-07206-2)
Supplement: Supplementary file 1 — Additional file 1. Trial Protocol. [file 13063_2023_7206_MOESM1_ESM.pdf]

Clinical Effectiveness of the ‘PICU Up!’ Multifaceted Early Mobility Intervention for Critically Ill Children: A pragmatic, stepped-wedge trial

Supported by:
The Eunice Kennedy Shriver National Institute of Child Health and Human Development, USA

Principal Investigator & Protocol Committee Chair:
Sapna R. Kudchadkar, MD, PhD, FCCM
Associate Professor
Department of Anesthesiology and Critical Care Medicine
Department of Pediatrics
Department of Physical Medicine and Rehabilitation
Johns Hopkins University School of Medicine

Protocol Committee:

Dale Needham, MD, PhD	Johns Hopkins University School of Medicine
Ayse Gurses, PhD, MS, MPH	Johns Hopkins University School of Medicine
Elizabeth Colantuoni, PhD	Johns Hopkins Bloomberg School of Public Health
Michele Balas, PhD, RN	University of Nebraska Medical Center College of Nursing
Victor Dinglas, MPH	Johns Hopkins University School of Medicine
Colleen Mennie, RN	Johns Hopkins University School of Medicine

Protocol Revision History

Version Number: 2.0
Version Date: 2/20/2023
Summary of Revisions Made:

Version 1.1:

None, new protocol

Version 2.0:

Additional language describing the interim analysis plan
Additional details in the definition of outcomes

Statement Of Compliance

The study will be carried out in accordance with Good Clinical Practice (GCP) as required by the following:

United States (US) Code of Federal Regulations (CFR) applicable to clinical studies (45 CFR Part 46; 21 CFR Part 50, 21 CFR Part 56, and 21 CFR Part 312)

ICH E6; 62 Federal Register 25691 (1997)

All key personnel (all individuals responsible for the design and conduct of this study) have completed Human Subjects Protection Training.

Abbreviations

AE	Adverse Event
ACF	Alive and coma free
ADCF	Alive and delirium and coma free
AVF	Alive and ventilator free
CCC	Clinical Coordinating Center
CONSORT	CONsolidated Standards of Reporting Trials
CRF	Case Report Form
DCC	Data Coordinating Center
DSMB	Data Safety Monitoring Board
ECMO	Extracorporeal membrane oxygenation
EHR	Electronic Health Record
ERIC	Expert Recommendations for Implementing Change
IRB	Institutional Review Board
JHM	Johns Hopkins Medicine
LOS	Length of stay
OT	Occupational Therapist
PCPC	Pediatric Cerebral Performance Category
PICS	Post-Intensive Care Syndrome
PICU	Pediatric Intensive Care Unit
POPC	Pediatric Overall Performance Category
PT	Physical Therapist
QA	Quality Assurance
QI	Quality Improvement
RCT	Randomized Control Trial
SAE	Serious Adverse Event
SBS	State Behavioral Scale
SEIPS 2.0	Systems Engineering in Patient Safety 2.0
sIRB	Single Institutional Review Board
SUSAR	Suspected, unexpected, serious adverse reaction
TSW	Traditional stepped-wedge

Contents

	Page
**Protocol Revision History**	1
**Statement Of Compliance**	2
**Abbreviations**	3
**Study Team Roster**	5
**Participating Study Sites**	6
**Précis**	7
**1. Study Objectives**	8
1.1 Primary Objective	8
1.2 Secondary Objectives	8
**2. Background and Rationale**	9
2.1 Background on Condition, Disease, or Other Primary Study Focus	9
2.2 Study Rationale	9
**3. Detailed Description of the Trial Methods**	12
3.1 Study Design and Population	12
3.2 Implementation of the Intervention	18
3.3 Patient-Level Outcomes	21
**4. Process Evaluation Methods**	24
4.1 Overview of Process Evaluation	24
4.2 Detailed Process Evaluation Methods	24
4.3 PICU Up! Intervention Performance	25
**5. Statistical Plan and Analytic Approach**	27
5.1 Power and Sample Size for Patient-Level Outcomes	27
5.2 Analytic Plan for Patient-level Outcomes	27
5.3 Analytic Plan for Process Evaluation	28
5.4 Interim Analyses	29
**6. Human Subjects**	30
6.1 Institutional Review Board Reliance and Oversight	30
6.2 Risks to Human Subjects	30
6.3 Adequacy of Protection Against Risks	32
6.4 Protocol Deviations and Violations	33
6.5 Potential Benefit of the Research to Participants and Others	34
6.6 Importance of the Knowledge to be Gained	34
**7. Safety Monitoring**	35
**8. Publication Of Research Findings**	37
**9. References**	38

Study Team Roster

Principal Investigator

Sapna R. Kudchadkar, MD, PhD	sapna@jhmi.edu

Clinical Coordinating Center

Sapna R. Kudchadkar, MD, PhD	
Colleen Mennie, RN	cmennie1@jhmi.edu
Razvan Azamfirei, MS	razvan@jhmi.edu

Data Coordinating Center

Dale Needham, MD, PhD	dale.needham@jhmi.edu
Elizabeth Colantuoni, PhD	ejohnso2@jhmi.edu
Victor Dinglas, MPH	victor.dinglas@jhmi.edu

Process Evaluation

Ayse Gurses, PhD, MS, MPH	agurses1@jhmi.edu
Michele Balas, RN, PhD	balas.17@osu.edu
Sapna R. Kudchadkar, MD, PhD	

Participating Study Sites

Table 1: Characteristics of Participating Sites

Institution	Hospital Name	Site PI	Email Address
Baylor College of Medicine	Texas Children's Hospital	Matthew Musick, MD	mamusick@texaschildrens.org
Dartmouth College Geisel SOM	Children's Hospital at Dartmouth	Kelly Corbett, MD	kelly.corbett@hitchcock.org
Geisinger Commonwealth	Janet Weis Children's Hospital	Elizabeth Scarlett, MD	eescarlett@geisinger.edu
Medical College of Wisconsin	Children's Hospital of Wisconsin	Charles Rothschild, MD	crothschild@mcw.edu
Stanford University	Valley Children's Hospital	Molly Dorfman, MD, MPH	mdorfman@valleychildrens.org
University of Central Florida	Nemours Children's Hospital	Mashael Alqahtani, MBBS, MS	mashael.alqahtani@nemours.org
University of Louisville	Norton Children's Hospital	John Berkenbosch, MD	john.berkenbosch@louisville.edu
University of Minnesota	Hennepin Healthcare	Andrew Kiragu, MD	andrew.kiragu@hcmed.org
University of North Carolina	UNC Children's Hospital	Tracie Walker, MD	twalker3@med.unc.edu
West Virginia University	WVU Med Children's Hospital	Mel Wright, DO	mwright@hsc.wvu.edu

Précis

Study Title
Clinical Effectiveness of the ”PICU Up!” Multifaceted Early Mobility Intervention for Critically Ill Children: A pragmatic, stepped-wedge trial

Short Title: The PICU Up! Stepped-wedge Trial

Objectives:

1. To evaluate if the PICU Up! intervention, delivered in real-world conditions, decreases mechanical ventilation duration (primary outcome) and improves delirium and functional status compared to usual care in critically ill children.

2. Conduct a multi-stakeholder, mixed-methods process evaluation to identify key contextual (work system) factors associated with delivery of PICU Up!.

Design, Interventions, and Duration This study is a mixed-methods, stepped-wedge, cluster randomized trial of a pragmatic, interprofessional, and multifaceted early mobility intervention (PICU Up!) conducted in 10 hospitals with a primary outcome of mechanical ventilation duration, defined as days alive and ventilator free (AVF), and secondary outcomes of delirium and functional status. Over a 2-year period, data will be collected on 1,440 PICU patients.

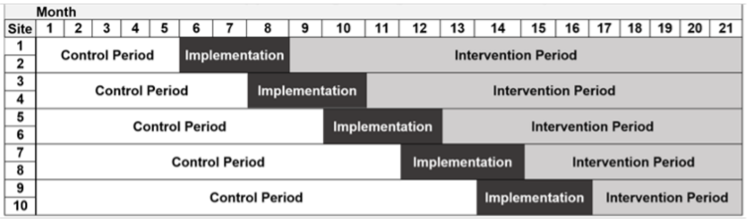

The study includes an embedded process evaluation (to be added to the protocol in subsequent amendments) to identify factors associated with reliable PICU Up! adoption and performance. All participating PICUs will simultaneously start the control period with data collection and conduct implementation planning for the PICU Up! intervention, a multifaceted, interprofessional, and systematic tiered pathway that is integrated into routine PICU practice to safely optimize early and progressive mobility. The order in which the PICUs will move into the intervention period will be determined through randomization. Data collection will occur only during the control and intervention phases. Data collection includes patient demographic, clinical, and rehabilitation data from the Epic electronic health record (EHR) from PICU admission through Day 21 or PICU discharge, whichever comes first.

Sample Size and Population As a pragmatic trial, *all* PICU patients regardless of their length of stay are screened for the PICU Up! unit-based intervention, which includes criteria for exclusion based on specific clinical factors (i.e., open chest or abdomen, ECMO).[1] Criteria for inclusion in data collection and statistical analysis are children receiving invasive mechanical ventilation ≥ 48 hours on Day 3 of PICU admission (n=1,440).

1 Study Objectives

1.1 Primary Objective

To evaluate if the PICU Up! intervention, delivered in real-world conditions, decreases mechanical ventilation duration (primary outcome) and improves delirium and functional status compared to usual care in critically ill children. Our hypothesis is that PICU Up! (compared to usual care) will shorten the duration of mechanical ventilation resulting in an increase of at least 1.8 days AVF during the 21-day follow-up.

1.2 Secondary Objectives

a. To evaluate if the PICU Up! intervention, delivered in real-world conditions improves:

    i. Delirium, defined as days alive and delirium- and coma-free (ADCF)

    ii. Functional status at the earlier of PICU discharge or PICU Day 21 (Pediatric Cerebral Performance Category and Pediatric Overall Performance Category)

    iii. Exploratory Outcomes: Deep sedation (defined as days alive- and coma-free, ACF), PICU length of stay (through Day 21), PICU mortality (through Day 21), PICU and hospital disposition (discharge to home/inpatient floor/inpatient rehabilitation/other hospital), new pressure injury (through Day 21), and opioid and benzodiazepine exposure (morphine- and benzodiazepine-equivalents median mg/kg/day).

b. To evaluate the proportion of PICU Up! elements performed as a measure of reliable PICU Up! delivery.

2 Background and Rationale

2.1 Background on Condition, Disease, or Other Primary Study Focus

Over 250,000 children are admitted to U.S. Pediatric Intensive Care Units (PICUs) annually, with 25% of PICU patients utilizing 75% of all bed-days.[2, 3] As such, the number of U.S. PICU beds has increased by 43% since 2000.[2, 4] PICU mortality rates have decreased by over 50% in the last 2 decades due to the changing spectrum of pediatric critical illness and medical and technological advances.[5, 6] However, decreased PICU mortality has led to a growing number of survivors experiencing both short and long-term morbidities.[5, 7, 8] Importantly, over half of all critically ill children develop preventable PICU-acquired morbidity, including iatrogenic opioid or sedative withdrawal, delirium, venous thromboembolism, pressure injury, and ICU-acquired muscle weakness, with the highest risk in mechanically ventilated patients.[9-18] These morbidities are strongly associated with poor functional recovery, which leads to poor quality of life and increased parental stress.[9, 19] The resulting long-lasting physical, psychological, and neurocognitive impairments are known as pediatric post-intensive care syndrome (PICS).[5, 7, 8, 20] The recognition of PICS has resulted in substantial research in adult ICU populations, and is recognized by the NIH/NHLBI and major academic societies as a high research priority.[21-25] Thus, the preventable morbidities experienced by a growing number of PICU survivors are an important public health issue urgently in need of evidence-based strategies.

2.2 Study Rationale

Why will a multifaceted, interprofessional intervention to increase mobility improve PICU patients' outcomes?

a. *Early and Progressive Mobility in the PICU*

Early (within 72 hours of ICU admission) and progressive mobility is associated with improved outcomes in critically ill adults including shortened duration of mechanical ventilation and improved muscle strength.[26-40] However, the clinical effectiveness of early and progressive mobility in the PICU has never been rigorously studied. Unlike adults, critically ill children are admitted to the PICU during a crucial period of physical and neurocognitive development. Less than 40% of PICU survivors with normal baseline function recover to their baseline by 3 months after PICU discharge.[9] Moreover, our multicenter point prevalence study of physical rehabilitation in 82 U.S. PICUs found that 68% of all admissions ≥ 3 days are patients ≤2 years old.[41] Thus, there is an urgent need to attend to both rehabilitation and to habilitation, the acquisition of new physical and cognitive skills, in the PICU. Our research and others have shown that the youngest children and those with normal baseline function have the longest delays to starting mobilization, likely due to a misperception by the medical team that they are at lower risk for functional deterioration.[9, 41, 42, 43] Physical and occupational therapists (PT and OT) have the expertise to address PICU patients' physical and cognitive needs across the age spectrum.[44] Yet, our data shows that 30% of PICU patients do not have either a PT or OT consult by Day 10 of admission.[41]

The focus of early PICU care is on resuscitation and stabilization of acute disease processes. Thus, any form of mobility is often considered only after the child has begun to recover and is not "too sick to mobilize".[45]
Therefore, the scientific premise is that the clinical effectiveness of early and progressive mobility to improve outcomes must be evaluated using a rigorous, pragmatic, interprofessional, and unit-based approach.

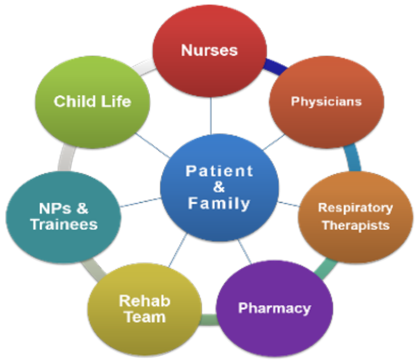

Figure 1: Team Model for Early Mobility in the PICU

b. Interprofessional and Family Teamwork to Promote Mobility

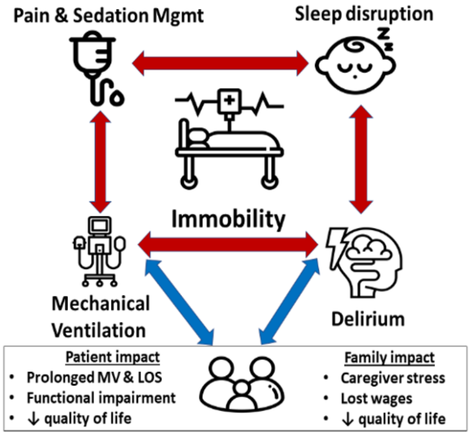

Figure 2: Conceptual framework guiding PICU Up! design

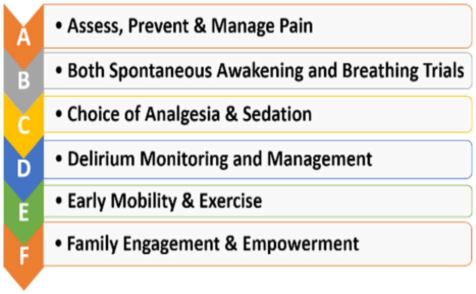

Figure 3: ABCDEF Bundle

Pediatric rehabilitation team resources are finite in the critical care setting, with <20% of U.S. PICUs having a dedicated full-time equivalent PT or OT.[41] Optimizing mobility in complex PICU patients requires effective interprofessional shared decision-making and teamwork (i.e., coordination, communication, collaboration) from all disciplines involved in their care (Figure 1).[1, 46] Nurses and families are the foundation of mobility in the PICU. Nurses are at the bedside 24 hours a day. Furthermore, 100% of the 82 U.S. PICUs in our rehabilitation point prevalence study allow 24-hour family presence at the bedside.[41] As such, PICU nurses facilitate 67% of all mobilization activities alone or in combination with family or other staff, while only 14% are therapist-provided mobility.[41] In preliminary data from our qualitative study, nurses felt their participation in patient mobility was beneficial to the patient and personally rewarding if they were given the educational tools to safely mobilize patients and had PT/OT collaboration. Families are also highly supportive of mobility,[47] but need guidance on how they can help through effective communication with the PICU team.[48, 49, 50]
Engaging families in their child's care and considering them as part of the care team is key to effective family-centered care.[51, 52] Therefore, the scientific premise is that clinical effectiveness of early mobility interventions will be optimized with interprofessional shared-decision making and family collaboration.

c. Multifaceted Intervention to Promote Early and Progressive Mobility

Despite known harms of bedrest, a PICU culture of immobility is facilitated by oversedation, physical restraints, and poor sleep hygiene due to a perceived need to optimize safety and comfort.[53, 54, 55] These factors all increase the risk of prolonged mechanical ventilation.[56] Single-component approaches to decrease duration of mechanical ventilation in the PICU have not consistently shown effectiveness.[57, 58, 59, 60] The largest multicenter RCT of protocolized sedation for children with acute respiratory failure led to no difference in ventilation-free days.[57]

d. Pain and sedation management, sleep hygiene, delirium, extubation readiness assessments, mobility and family engagement are all intimately interconnected in ICU care

A child who is deeply sedated, for instance, is more likely to be delirious, with higher risks for mobilization and lower likelihood of extubation readiness. Our data suggest that family members are less likely to engage when their child is sedated and mechanically ventilated.[61] We have also shown that sleep is severely disturbed in the PICU,[55, 62, 63] and poor sleep can increase delirium and impact participation in mobility**(Figure 2)**.[64] These issues have been mitigated in adult ICUs with early, exercise-based rehabilitation as part of the interprofessional ABCDEF Bundle **(Figure 3)**. [65, 66] The ABCDEF bundle is associated with lower mortality, less delirium and more ventilator-free days in critically ill adults.[65-68] However, the heterogeneity of the PICU patient population limits direct translation of this approach and has never been rigorously studied.

3 Detailed Description of the Trial Methods

3.1 Study Design and Population

a. Study Design

The study will be conducted using a pragmatic stepped-wedge cluster randomized trial design, with each PICU acting as one cluster. This study protocol is reported in accordance with the CONSORT (CONsolidated Standards Of Reporting Trials) extension for stepped-wedge cluster RCTs guideline.[69-71] In a traditional stepped-wedge (TSW) design, all clusters begin in the control group and then transition to the intervention group at sequential and randomly assigned periods, facilitating the delivery of a desired intervention to all clusters, in this case, all participating PICUs.
There are several additional advantages to a TSW trial compared to a traditional cluster RCT including:

i. TSW design overcomes the logistical constraint of not being able to implement and deliver the intervention simultaneously to all PICUs.

ii. The TSW design is ideal for interventions that are designed to be implemented on a unit level, and for which patient-level randomization may result in contamination between control and intervention groups.[71]

iii. In contrast to the before-and-after design utilized for our single-center QI study,[1] the TSW allows for a robust comparison between treatment and control periods by accounting for temporal trends in the data.

After IRB approval and setup for data collection, all participating PICUs will simultaneously start the control period with data collection and continue implementation planning as described in **Section 3.2.b**. The order in which the PICUs will move into the intervention period will be determined through randomization. Data collection (**Section 3.1.f**) will occur only during the control and intervention phases. **Table 2** displays the trial's TSW design.

b. Randomization Strategy

Site \ Month	1	2	3	4	5	6	7	8	9	10	11	12	13	14	15	16	17	18	19	20	21
1, 2	Control Period					Implementation			Intervention Period												
3, 4	Control Period							Implementation			Intervention Period										
5, 6	Control Period									Implementation			Intervention Period								
7, 8	Control Period											Implementation			Intervention Period						
9, 10	Control Period													Implementation				Intervention Period			

Table 2: Stepped-Wedge Design for the PICU Up! Trial

Randomization will occur at the PICU level. The role of randomization in stepped-wedge trials is to 1) determine the order in which the clusters (PICUs) will transition from control to intervention; 2) increase internal validity of the study; and 3) increase the transparency and perceived fairness of allocation.[72] Two PICUs will cross over to the intervention at a time (5 steps). Since there is only one PICU participating in each hospital, we will not need to account for contamination between PICUs in the same hospital. All possible permutations will be considered for the order of transition from control to intervention. However, some

permutations will lead to the largest PICUs transitioning early and the smallest PICUs transitioning late, or vice versa. In this case, there would be an imbalance in patients included in the control and intervention phases. Therefore, the imbalance for each permutation will be estimated based on anticipated PICU-specific eligibility expectations, and permutations with excessive imbalance will be excluded. The permutation selected for implementation will then be chosen at random from permutations with adequate balance. The R statistical package will be used to create the set of unique permutations, compute the imbalance score and finally sample from eligible permutations.[73] Each site study team will be notified of their site's transition date four months in advance. PICU Up! will then be implemented over three months including PICU staff training as outlined in **Section 3.2.b**. All PICUs will remain in the study for the entire period, with each pair of PICUs being exposed to the intervention for different durations based on the randomization schedule **(Table 2)**.

c. Study Setting

The PICU Up! trial will include 10 PICUs in 10 states within the U.S. (See **Participating Study Sites**). These sites [1] represent a diverse mix of public, private, and federally-funded teaching and nonteaching hospitals, with small to large ICUs, located in urban and suburban areas with varied patient populations (medical-surgical or medical-surgical-cardiac); and [2] do not have an existing PICU mobility protocol and would commit to not implementing a mobility protocol until their randomized time of implementation; All sites have agreed to engage in a single IRB reliance agreement with Johns Hopkins University. Across all study sites, Dr. Kudchadkar (PI) will lead efforts to train clinical teams to adapt and implement the PICU Up! intervention locally. All participating sites utilize the Epic Electronic Health Record (Epic Systems Corporation).

Table 3: Inclusion and Exclusion for Data Collection

Inclusion Criteria	Exclusion Criteria	Rationale for Exclusion
Invasive mechanical ventilation via oral/nasal endotracheal tube	Active or anticipated withdrawal of life support w/in 48h	Patients unlikely to receive benefit from PICU Up!
Mechanical ventilation ≥48 hours at 7 a.m. on PICU Day 3	Open chest or abdomen	Exclusion Criteria for PICU Up!
	Extracorporeal Membrane Oxygenation (ECMO)	Exclusion Criteria for PICU Up!

d. Study Population

As a pragmatic trial, all PICU patients, regardless of their length of stay, are screened for the PICU Up! unit-based intervention, which includes criteria for exclusion based on specific clinical factors (i.e. open chest or abdomen, ECMO).[1] Criteria for inclusion in data collection and statistical analysis **(Table 3)** will focus on children who are receiving

invasive mechanical ventilation ≥ 48 hours on Day 3 of PICU admission for which we expect our intervention will have the highest impact. This inclusion criterion will ensure that children who are only mechanically ventilated in the perioperative period or for short periods (procedures, transport) are not included. If the patient is readmitted to PICU during the index hospitalization, they will be included in data collection if they once again meet inclusion criteria on the subsequent admission with repeated outcome assessment as outlined in C.1.i. (n=1,440 patients)

e. Informed Consent

All eligible subjects at participating sites will have data collected under a waiver of informed consent. The Johns Hopkins IRB has granted a waiver of consent in our pilot multicenter PICU Up! trial based on the factors below. Full details are included in the section entitled **Adequacy of Protection Against Risks** (**Section 6.3**)

i. The intervention is targeted to the PICU care environment and interprofessional shared-decision making and does not deviate from accepted clinical practice. All mobility activities and goals are individualized and determined solely by the patient's medical team and not the PICU Up! intervention.

ii. The PICU Up! intervention poses no more than minimal risk, because it is a unit-based intervention focused on PICU workflow and interprofessional shared-decision making. Sedation goal-setting, delirium screening, sleep promotion, rehabilitation team consultation, extubation readiness assessments, family engagement and mobilization activities are already practiced in PICUs and these processes will be at least equivalent, or improved, with the PICU Up! Intervention.

iii. The PICU Up! intervention involves no procedures for which written consent is normally required outside of the research context, and this research could not practicably be carried out without a waiver of consent. All patients and families in the participating PICUs will be made fully aware of the unit-wide PICU Up! intervention when the PICU is randomized to the implementation phase through patient-family education by local PICU staff.

iv. The waiver will not adversely affect the rights and welfare of the subjects. All data will be deidentified for transmission to the Data Coordinating Center (DCC). Protections against risks, including the risk for breach of data confidentiality, are delineated below.

v. Obtaining informed consent would threaten the scientific validity of the study, which depends on capturing all eligible patients during the enrollment period.

vi. Whenever appropriate, patients and their families will be provided with additional pertinent information after participation.

f. Data Collection

When the patient meets eligibility as outlined in **Table 3**, demographic, clinical and rehabilitation data will be collected from the Epic electronic health record (EHR) from PICU

admission through Day 21 or PICU discharge, whichever comes first. Data collection variables/timing are summarized in **Table 4**. This data collection schedule has been comprehensively tested for feasibility in our multicenter pilot trial. Daily data collection during invasive mechanical ventilation takes, on average, 12 minutes per patient per day. We chose 21 days for the duration of data collection for both feasibility and clinical relevance based on our pilot data.

Table 4: Data Collection Schedule

**ADMISSION DATA COLLECTION (ONE TIME)**	**Variable Type**
Demographic data (age, sex, race)	Continuous/ Categorical
Height weight; Body Mass Index	Continuous
Baseline Function: Pediatric Overall Performance Category[53,74] & Pediatric Cerebral Performance Category[74]	Ordinal
PICU Admission Diagnosis (e.g. cardiac, respiratory, sepsis, surgical)	Categorical
Severity of Illness: Pediatric Risk of Mortality Score IV[67, 75, 76]	Continuous
**INVASIVE MECHANICAL VENTILATION DAYS ONLY (DAILY THROUGH 21)**	
Mode of Ventilation (Conventional, High Frequency Oscillatory Ventilation, High Frequency Jet Ventilation)	Categorical
FiO_2 (%)	Continuous
Analgesic and Sedative Medications Doses (morphine milligram equivalents (MME) and midazolam miligram equivalents/kg/day)	Continuous
State Behavioral Scale(SBS):[77] daily SBS goal, number of documented sedation assessments/day & range	Continuous
Extubation Readiness Testing Outcome	Categorical
**ALL PATIENT DAYS THROUGH DAY 21 or PICU DISCHARGE (DAILY)**	
Respiratory Support (room air, supplemental O_2, Heated HFNC, CPAP, BiPAP, Trach collar, Mechanical ventilation via tracheostomy, Invasive mechanical ventilation)	Categorical
Minimum activity goal & highest level of mobility achieved (adapted Johns Hopkins Highest Level of Mobility Scale)[78]	Ordinal
Documentation of therapy from the rehabilitation team (Physical Therapy, Occupational Therapy, Speech Language Pathologist)	Categorical
Pain assessment: # of documented assessments/day using validated tools & range	Continuous
Delirium assessment: number of documented assessments (Cornell Assessment of Pediatric Delirium[79]) and score	Continuous
Family engagement: Documentation of family visitation/engagement on rounds/participation in mobility (yes/no)	Categorical
PICU Up! Level (intervention phase only)	Ordinal

Continued on next page

Table 4: Data Collection Schedule (Continued)

Safety event: fall, unplanned extubation or device removal, cardiac arrest	Categorical
New pressure injury	Categorical
**DISCHARGE DATA COLLECTION (ONE TIME)**	
Reason for completion: PICU discharge/death/exclusion criteria	Categorical
Discharge function: Pediatric Overall Performance Category[53, 74] & Pediatric Cerebral Performance Category[74]	Ordinal
Discharge Disposition (Inpatient Floor, Inpatient Rehab, Home, Transfer to another PICU)	Categorical

g. Blinding and Minimizing Bias

It is not possible to blind this study to patients, families, clinicians or research staff. However, our primary outcome (Days Alive and Ventilator Free) is objective and quantitative and not likely biased by care team members' perceptions or behaviors.

h. Data Coordinating Center

This study's Data Coordinating Center (DCC) will employ a multifaceted approach to data quality control.[80] First, the DCC will provide centralized training and data quality assurance to each site as outlined herein:

1) the DCC will maintain an online repository of the current and prior versions of the study protocol, case report forms, manuals of operations and other training materials;
2) data collection will be protocolized, and staff trained and re-trained on protocols with initial and ongoing quality assurance procedures; and
3) the DCC will conduct periodic data cleaning, including review of descriptive statistics of data and logic checks of the data during the study with regular data queries and PICU Up! performance feedback to sites.

The DCC will utilize REDCap, a web-based database, to capture, manage, and export research data using unique de-identified research participant identifiers.[81] The REDCap database features secure, HIPAA-compliant data storage as well as an audit trail for all data field edits. Each site will only have access to their own data and only authorized IRB-approved personnel will receive database access. The REDCap database will include automated data validation, range checks, and missingness checks at entry. Data use agreements will be established between all sites and the DCC. The DCC will transmit data, as needed, via encrypted transfer.

i. Study Leadership and Management of PICU Up! Trial

To address important leadership, communication, implementation or quality issues, our Steering Committee (PI, 4 Co-Is, CCC Program Manager and DCC Research Manager) will meet at least monthly (weekly, as needed) by phone to discuss day-to-day issues arising during trial conduct. We will have a communications plan to ensure constant, consistent, and important information is reaching key stakeholders in a timely fashion. Each site PI will supervise day-to-day local operations in close contact with the site's PICU Up! Champion team and research staff (see **Study Team Roster**).

j. Clinical Coordinating Center

The Clinical Coordinating Center (CCC) will be led by Sapna Kudchadkar, MD, PhD, the Principal Investigator and Chair of the CCC, along with CCC Program Manager Colleen Mennie, RN. The CCC is responsible for oversight of study activities at all 10 participating sites, including the single IRB process, training and education for implementation of the PICU Up! Intervention, monitoring intervention fidelity and reporting of protocol events and deviations. The CCC will keep current contact information for all sites, and will ensure that each participating site has a Federalwide Assurance (FWA) for the Protection of Human Subjects on file with the Office for Human Research Protections (OHRP).

The CCC will maintain a study website that can only be accessed by approved study team members from each participating site. The website will contain the most current version of the protocol, amendments to the protocol, and educational and study related resources. These study materials will also be distributed via email to the site PI's and research coordinators as they are available or updated.

As part of the onboarding process, all participating site study team members will receive training from the CCC regarding protocol events and deviations as well as JHM IRB's prompt reporting requirements. Policy updates and refreshers will be provided throughout the entirety of the study. Each site will promptly report any protocol events or deviations through the study site's REDCap portal. This data collection platform (see **Section 3.1.h**) is monitored daily by both the CCC and the Data Coordinating Center (DCC), and the CCC will be responsible for ensuring appropriate reporting of these events and deviations to the JHM IRB. The DCC will be responsible for all data collection and management, including annual enrollment data (see **Section 3.1.f-h**).

3.2 Implementation of the Intervention

a. Description of the PICU Up! Intervention

PICU Up! is a multifaceted, interprofessional and systematic tiered pathway that is integrated into routine PICU practice to safely optimize early and progressive mobility. Detailed methodology for the development of the PICU Up! intervention has been published.[1] PICU Up! incorporates the screening process for determining a patient's appropriate activity level into the daily rounding workflow for all PICU patients, with a tiered activity plan (**Figure 4**) based on clinical parameters to individualize goals based on each child's unique needs. Included are criteria for pausing the activity and for reassessing the patient before continuing the activity.[1]

While the PICU Up! level is based on objective criteria, the interprofessional team collectively determines the daily activity goal(s) through shared decision-making, which is documented in the medical record on morning rounds. The intervention includes a rounding template which ensures daily discussion of the PICU Up! level (**Figure 4**) and elements: 1) analgesia; 2) protocolized extubation readiness testing; 3) sedation level and goal; 4) delirium screening; 5) mobility goal including PT/OT consultation by Day 3; 6) family engagement in mobility; and 7) sleep promotion. As a pragmatic trial, all other aspects of PICU care will be conducted per routine practice.

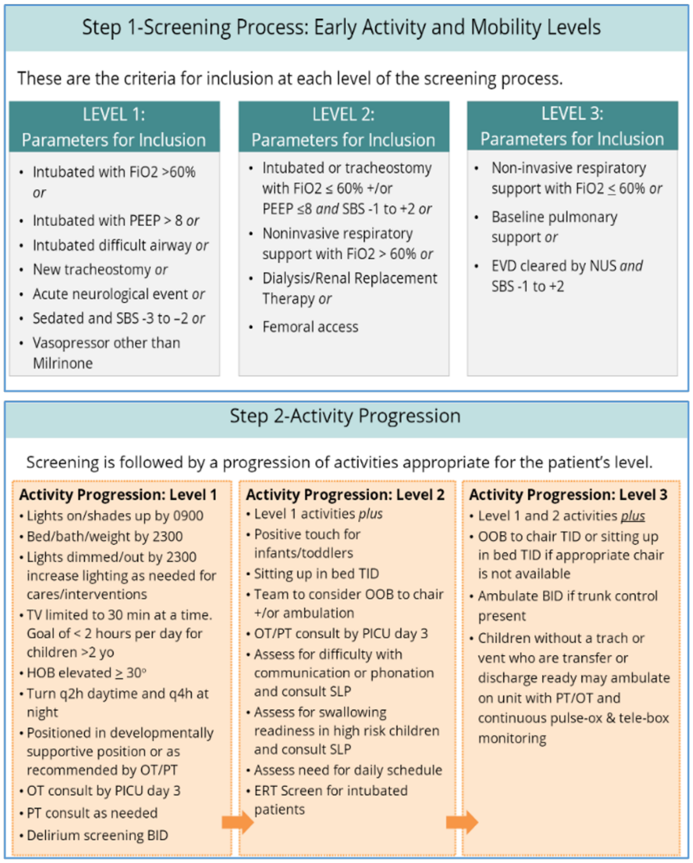

Figure 4: PICU Up levels and corresponding activity

b. PICU Up! Implementation Process at Participating Sites

The timeline and training materials for PICU Up! implementation outlined below have been comprehensively tested in our multicenter PICU Up! pilot stepped-wedge trial. During study setup, each site will build their PICU Up! Champion Team with at least 2 representatives from each relevant discipline. The CCC will conduct monthly video conference training with each of the Champion teams for PICU Up! implementation planning including integration of PICU Up! elements.[77, 79, 82] The initial site initiation webinar (Month 1) will include all champions and begin skills development to learn PICU Up! elements and strategies for rollout, along with orientation to all training materials/planning resources **(Table 5)**. Teams will coordinate local planning efforts after identifying local barriers and facilitators to implementation and will be informed of their randomized time for implementation 4 months in advance to allow for adequate preparation time. During each site's implementation, educational materials in multiple formats developed, utilized and refined from our single-center[1] and multicenter pilot study will be presented in meetings, discipline-specific webinars and online communications by the local team to ensure engagement of all PICU staff.

A key component of the educational program is a required 45-minute online learning module for all PICU staff about early rehabilitation in the PICU, including interactive case-based scenarios to illustrate application of the PICU Up! criteria. We have con-

firmed the compatibility of each site's e-learning platform with our module, which has been utilized in our single-center and multicenter pilot trial.No data collection will occur during the 3-month implementation phase to ensure the site team's focus on implementation and to allow for outcomes evaluation after implementation is complete. The education phase of implementation will be one month. In Month 2, the PICU Up! intervention will be deployed. Each child's activity level will be discussed and established during morning rounds, with the bedside nurse reporting the PICU Up! level based on the established criteria **(Figure 4)**. The PICU team will determine the daily activity goal during rounds. The bedside nurse will record this information in a templated electronic record daily goals note, accessible to all staff. Activities can be conducted by nursing and/or therapists based on their skillset and the child's needs. Families will receive PICU Up! education on admission.

Table 5: Implementation Planning Resources for Site Champions

**Site Champion Resources**	**PICU Up! Go-Live Materials (all PICU Staff)**
Monthly CCC training (video conference)	Online learning module w/illustrative cases
PICU Up! discipline-specific webinars	PICU Up! Patient-Family Handbook
Resource Library on key PICU Up! topics:<br>· Creating the Interdisciplinary Team<br>· Optimizing Sedation in the PICU<br>· Promoting Sleep Hygiene<br>· Delirium Screening in the PICU<br>· Implementation: Getting to "Yes"<br>· Family Engagement: A key resource<br>· PICU Nurses as Mobility Champions<br>· The Role of Child Life Specialists<br>· Which Equipment?<br>· Making PICU Mobility Fun	Bedside binders with SBS, delirium & activity resources
	PICU Up! pocket cards: Includes State Behavioral Scale and delirium screening guidance
	PICU Up! educational posters (staff & family)
Electronic Health Record (Epic) Templates<br>· PICU Up! Flowsheet<br>· Daily Rounding Note<br>· Extubation Readiness Trial Flowsheet	PICU Up! Policy Documents (for unit-specific adaptation)
	Rounding Script Template w/ PICU Up! Elements
PICU Up! Barriers and Facilitators Tool	PICU Up! Family Menu & info materials

3.3 Patient-Level Outcomes

Primary and Important Secondary Endpoints:

a. Primary Outcome: Days Alive and Ventilator Free (AVF) through Day 21.

We will consider Time 0 as the time of endotracheal intubation or PICU admission for patients intubated at an outside hospital and days free of ventilation are counted following the first time the endotracheal tube was continuously absent for at least 24 hours. Days AVF are counted for both patients who die prior to or survive through day 21. Patients will be assigned 0 days AVF if they remained intubated through 21 days or were transferred prior to day 21 without remaining extubated for more than 24 hours. We purposefully chose days AVF as the primary outcome given the abundance of literature suggesting the negative impact prolonged mechanical ventilation has on children’s outcomes and established association with sedative exposure, delirium, and other ICU-acquired morbidities. We chose days AVF instead of duration of mechanical ventilation due the challenge of analyzing duration of mechanical ventilation in the presence of patient mortality, which could introduce bias in our analysis if not appropriately accounted for. For example, standard survival analysis methods would treat patient mortality as an independent censoring event for duration of mechanical ventilation, even though these outcomes are likely correlated. Days AVF was chosen over the alternative composite “alive and ventilator free days” as days AVF considers any day off the ventilator as important to the patient, family, and provider regardless of whether the patient ultimately survivors or not; whereas the latter is more heavily focuses on mortality by assigning a value of 0 to all patients that die regardless of the duration of mechanical ventilation. Additionally, we chose this primary outcome in lieu of PICU length of stay due to capacity factors that may delay PICU discharge. Even if a patient is ready to be transferred to the inpatient floor, they may remain in the PICU not due to clinical necessity but due to a lack of inpatient beds. Transition to tracheostomy with mechanical ventilation from endotracheal tube will count as mechanical ventilation days. If the patient was successfully extubated and discharged before 21 days, the days after discharge will count as ventilator-free. For patients discharged to a long-term ventilation rehabilitation facility, if the discharge happens before day 21, the remainder of the days to day 21 will be considered mechanical ventilation days **(Table 6)**.

b. Secondary Effectiveness Outcomes

Secondary outcomes will include the following:

i. Days alive and delirium- and coma-free (ADCF), which will be supplemented with days alive- and coma-free (ACF) and days alive. If the patient was discharged from the PICU alive, all days following discharge will be considered days alive, delirium- and coma-free **(Table 6)**.

ii. Functional status at PICU discharge or Day 21 (Pediatric Cerebral Performance Category and Pediatric Overall Performance Category)

c. Exploratory Outcomes

Exploratory outcomes will include: PICU length of stay (through Day 21), PICU mortality (through Day 21), PICU and hospital disposition (discharge to home/inpatient floor/inpatient rehabilitation/other hospital), new pressure injury (through Day 21) and opioid and/or benzodiazepine exposure (median mg/kg/day; morphine/benzodiazepine equivalents).

d. Safety Endpoints

All patients will be closely monitored as part of routine ICU care. Each site will document the following events of interest and determine if they meet prespecified criteria for Suspected Unexpected Serious Adverse Reaction (SUSAR; see **Section 7.6**): unplanned extubation or device removal, falls, and cardiac arrest.

Table 6: Definition of Outcome Measures

**Primary Outcome**	
Days Alive and Ventilator-Free (AVF)	Days when the patient was alive, and an endotracheal tube was absent for 24 continuous hours during a calendar day [1,2] through 21 days
**Secondary Outcomes**	
Days Alive and Delirium- and Coma-Free (ADCF)	Days when the patient was alive, without delirium, and coma-free for 24 continuous hours during a calendar day [2-4] through 21 days
Days Alive and Coma-Free (ACF)	Days when the patient was alive, and coma-free for 24 continuous hours during a calendar day [2,4] through 21 days
Days Alive	Days when the patient was alive for 24 hours during a calendar day [5] through 21 days
Functional Status	Pediatric Cerebral Performance Category and Pediatric Overall Performance Category scores assessed at the earlier of PICU discharge or Day 21
**Exploratory Outcomes**	
PICU Length of Stay	Number of days on which the patient was physically present in the PICU rounded to the nearest higher day through 21 days
PICU Mortality	Number of patients deceased or withdrawn from the study for limitation of care orders or brain death evaluation / total enrolled patients
PICU and Hospital Discharge Destination	Number of patients discharged to home, inpatient floor, inpatient, rehabilitation, other hospitals / number of discharged patients
New Pressure Injuries	Rate of new pressure injuries / eligible patient days through 21 days
Opioid Exposure	Opioid dose (mg/kg/day) measured in morphine milligram equivalents (MME) [6]
Benzodiazepines Exposure	Benzodiazepine dose (mg/kg/day) measured in midazolam milligram equivalents [7]

[1] For patients with a tracheostomy, a ventilator-free day will be defined as a day when the patient is not receiving invasive mechanical ventilation for 24 continuous hours during a calendar day. Specifically, patients with a tracheostomy whose breaths are unassisted (i.e trach collar) will be considered ventilator-free; patients receiving assisted breaths (i.e. CPAP) will not be considered ventilator-free.

[2] Days following PICU discharge to 21 days will be considered days alive, ventilator-, delirium- and coma-free, with the following exceptions: days after PICU discharge will not be considered days alive for deceased patients, days after PICU discharge will not be considered days ventilator-free if the patient remains intubated at discharge.

[3] Delirium-free days are days when the patient is at risk of delirium (i.e. coma-free) and delirium was not present.

[4] Coma-free days are days when all documented sedation scores are above the following thresholds: SBS >= -1; RASS >= -3; COMFORT-B >= 10.

[5] For patients who were withdrawn from the study due to limitation of care orders or brain death evaluations, they will be considered deceased on the day death is pronounced.

[6] The following conversion will be used: Fentanyl: 10 mcg = 1 MME; Hydromorphone: 0.25 mg = 1 MME; Morphine 1 mg = 1 MME.[83]

[7] The following conversion will be used: Lorazepam: 0.5 mg = 1 midazolam milligram equivalents; Midazolam: 1 mg = 1 midazolam milligram equivalents.[84]

4 Process Evaluation Methods

4.1 Overview of Process Evaluation

We will perform a mixed-methods process evaluation of the PICU Up! trial using the United Kingdom's Medical Research Council's framework for evaluating complex interventions and guided by our Systems Engineering in Patient Safety 2.0 Framework (SEIPS 2.0; Preliminary Data).[85] Our goal is to gain a deeper understanding of what implementation and contextual factors affect PICU Up! intervention performance. Our process evaluation involves rigorous quantitative and qualitative methodology and multiple data types/sources (survey questionnaires, document analysis, routine monitoring data). Defining the implementation and contextual factors that affect PICU Up! performance will ultimately lead to the development of tailored approaches to increase systematic uptake of this evidence-based, interprofessional intervention into routine clinical practice.

4.2 Detailed Process Evaluation Methods

As illustrated in **Figure 5**, there are three core components involved in a process evaluation (implementation, mechanisms of impact, and context). These key components are informed by a description of the intervention (**Section 3.2**) and its causal assumptions and inform the interpretation of study outcomes (**Section 4.1**). Our project specifically focuses on implementation and context.

a. Implementation Factors

We define implementation as the process through which interventions are delivered and what is actually delivered in practice. For this evaluation, we will specifically focus on: 1) how much of the intervention was delivered (dose); 2) how delivery was achieved; and 3) what alterations were made to PICU Up! in order to achieve a better contextual fit.

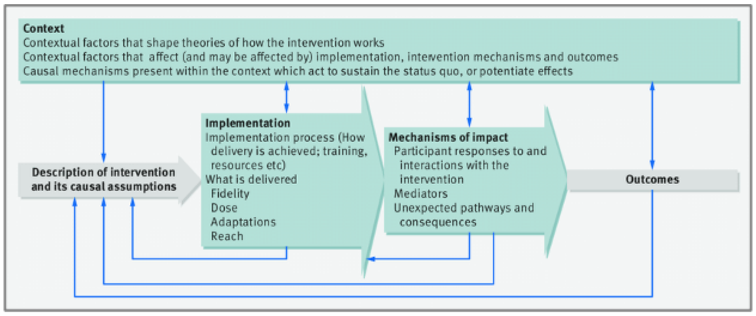

Figure 5: Key Functions of Process Evaluations and the Relationships amongst them[84]

Document Analysis

To determine if any alterations were made to the PICU Up! intervention in order to achieve a better contextual fit, we will perform a document analysis. We are specifically interested in learning if sites: 1) adapted/changed any of the early mobility screening process criteria or activity progression steps; 2) utilized and/or adapted any of the provided EHR templates (PICU Up! flowsheet, daily rounding note, extubation readiness); and/or 3) revised pain, sedation, delirium, mechanical ventilation, mobility, or sleep protocols/policies. To achieve this goal, we will ask all participating sites to submit copies of all PICU Up! related documentation forms and policies at study initiation and end of the trial data collection phase in Aim 1. These forms will be compared and differences within and between sites will be noted.

b. Contextual Factors

We define context as factors external to the intervention which may influence its implementation. For this evaluation, we are most interested in exploring how the work system factors illustrated in our SEIPS 2.0[85] framework (Preliminary Data) influence PICU Up! delivery and adoption.

Organizational & Resource Availability Survey
This online survey has been utilized and adapted from previous studies led by Dr. Kudchadkar (PI)[41] and Dr. Balas (co-I).[66, 68] The PICU Organizational and Resource Availability Survey will collect information on hospital and ICU organizational characteristics, utilization rates, staffing patterns/ratios, and rounding and PICU Up! practices. The first section contains questions on characteristics of the hospital (type, locale, teaching status) and PICU (type, training programs). The next section contains focuses on utilization data, such as total number of hospital/PICU/step-down beds and annual hospital/PICU admissions data. The third section collects PICU physician/advanced practice provider/RN/other ICU team member staffing, education, and certification data. The fourth section details ICU organization, such as whether it is an open, semi-open, or closed unit, and leadership structure. The final section has questions regarding PICU rounding and PICU Up! practices. This section collects specific data regarding daily rounds (time, location, attendees) use of pain, sedation, and delirium assessment tools, family engagement, which protocols and teamwork tools the PICU utilizes, and types/length of QI experience. This survey takes approximately 20 minutes to complete and will be filled out by the Site PI in collaboration with the PICU Nurse Manager and rehabilitation team leaders. The survey will be administered at the beginning of the study and end of the data collection period to determine of changes occurred.

4.3 PICU Up! Intervention Performance

Similar to the patient-level measures of PICU Up! performance in Aim 1, we will summarize the outcome of PICU Up! performance at the PICU level for each month of the intervention period. Specifically, we will calculate a measure of complete, proportional and individual element performance for each PICU. The individual PICU Up! elements and operational definitions are provided in **Table 7**. The monthly complete and proportional performance will respectively be defined as the proportion of patient-days in which every eligible element of PICU Up! was performed and the proportion of eligible elements received divided by the number of eligible elements. Individual performance will be defined as the proportion of patient-days where the individual element was received divided by the number of days where the individual element was eligible. Patient-days will only be included if the patient was in the PICU for the full 24 hours.

Table 7: Definition of PICU Up! Performance Measures[66]

Element	Days eligible	Performance
**A**	All days	≥ 6 pain assessments/24 hours using a validated pediatric pain scoring scale
**B**	Days when receiving invasive mechanical ventilation	Screened for extubation readiness trial once daily if receiving invasive mechanical ventilation
**C**	Days when receiving sedation and/or invasive mechanical ventilation	≥ 6 sedation assessments/24 hours using a validated sedation scale and sedation goal documentation if receiving sedation and/or invasive mechanical ventilation
**D**	Days when patient is at risk for delirium	≥ 2 delirium screening assessments using a validated pediatric delirium screening tool
**E**	All days	Documentation of mobility goal AND highest level of mobility achieved each day
**F**	All days	A family member was educated on PICU Up! and/or participated in rounds/conferences/ mobility

5 Statistical Plan and Analytic Approach

5.1 Power and Sample Size for Patient-Level Outcomes

Utilizing site-specific admissions data, PARK-PICU screening and clinical data, and data from our pilot multicenter trial, we anticipate that conservatively an average of 8 patients/cluster/month will be eligible for inclusion in data collection (mechanically ventilated ≥48h on PICU Day 3 and meeting rest of eligibility criteria). In the stepped-wedge design proposed, with two hospitals switching at each step, there will be a total of 180 months of observation (90 months control/90 months intervention). Thus, there will be 720 patients included in the baseline period and 720 patients included in the intervention (post-implementation) period. In stepped-wedge designs, the power calculation must incorporate the clustered nature of the design and the confounding effect of time.[71] Using preliminary data obtained from our multicenter pilot trial,[86] we simulated 1000 hypothetical stepped-wedge trials allowing the days AVF to day 21 to decline linearly over time (Month 1 mean (variance) of 13.2 (26.4) days AVF to day 21, with monthly decline of 0.1 days) with a fixed decline (i.e. effect of intervention is constant over time) at both the start of the implementation period and during the intervention period. Based on our preliminary data, we assumed that patients within hospitals will be correlated with an ICC of 0.01. Given the design, the anticipated sample size and a 5% Type I error rate, we will have 80% power to detect at least a 1.8 day improvement in the mean days AVF to day 21 comparing the intervention and control period. Current literature indicates a 1 to 2-day reduction in duration of mechanical ventilation is clinically meaningful.[57]

5.2 Analytic Plan for Patient-level Outcomes

All analyses for Aim 1 will be designed and overseen by Dr. Colantuoni at the DCC, in consultation with Dr. Kudchadkar (PI) and Dr. Needham (co-investigator). Descriptive statistics for continuous variables will be presented as the mean ± standard deviation and quantiles and categorical variables will be expressed as percentages. The effect of the PICU Up! intervention on the clinical outcomes will be estimated using mixed-effects models that include indicators for both the implementation (3-months in duration) and intervention vs. control periods and incorporate features of the stepped-wedge design by including a random intercept defined for each site to account for clustering of outcomes within sites and a fixed effect of time (month, modeled as a natural cubic spline) to account for temporal trends in the outcomes over the period of the study.[87] Linear mixed-effects models will be used for the primary outcome where the coefficient for the indicator of intervention vs. control period directly estimates the mean difference in days AVF by day 21 comparing the intervention and control period. To account for potential violations of model assumptions (e.g. non-constant variance and the possibility of more complex correlation patterns among patients over time from the same PICU), robust variance estimates will be used. Similar linear mixed-effects models will be used for the secondary outcomes of delirium (days ADCF by day 21), with supplemental outcomes days ACF and days alive, as well as PICU length of stay. We expect that the distribution of the primary outcome and the aforementioned secondary outcomes will be skewed; however, given the large sample size, the linear models should be robust for estimating the

mean difference in these outcomes. Poisson log-linear mixed-effects models with the same random and fixed effects will be used as a sensitivity analysis for the linear mixed-effects models proposed above. Logistic mixed-effects models will be used for binary patient-level outcomes (e.g. PICU mortality). For the ordinal outcomes, Pediatric Cerebral Performance Category and Pediatric Overall Performance Category, if sufficient data is present in each ordered category or if we collapse the 6 ordered categories into at least 3 categories, we will utilize multinomial models to evaluate the effect of the intervention. If the data suggest collapsing the ordered categories into only two categories, then binomial regression models will be used. The primary analysis for all outcomes will be unadjusted, that is, we will assume that patients enrolled in the study over time will be similar with respect to characteristics associated with the outcomes (e.g. age and severity of illness). Secondary analyses will include exploratory analyses to evaluate this assumption and the models described above will be extended to include adjustment for key patient characteristics (e.g. admission diagnosis, baseline function). We will plan to conduct analyses to detect significant differences in intervention effect among sex/gender. Additional secondary analyses will fit the models described above including adjustment for the patient-specific measure of PICU Up! performance by separately including the measure of proportional performance (ranging from 0 to 1) and the binary indicator of complete performance. Statistical significance will be set at p-value < 0.05 using a two-sided hypothesis test for the primary outcome analysis; all secondary analyses will be considered exploratory with two-sided 95% confidence intervals reported. We will conduct missing data assessment/estimation, and if necessary, employ methods for imputation of missing data using MICE in R.[88]

5.3 Analytic Plan for Process Evaluation

Online survey data will be summarized using descriptive statistics. The mean and standard deviation will be reported for continuous measures and frequency and percentages for categorical variables. Trends in performance within a PICU over time will be explored using spaghetti plots with locally weighted regression smoothers to explore the general trends over time across all PICUs. Mixed-effects Poisson models will be used to model the trends in performance over time via restricted cubic splines with a minimum of a random intercept defined at the PICU level (i.e. the models may include random slopes at the PICU level). Subsequently, the models will include the hospital and PICU-level covariates (organizational survey), such as structural characteristics, staffing patterns, organizational traits (e.g., open vs. closed ICU), rounding practices, and use of daily teamwork tools. Including these covariates will allow us to determine if variation in performance across the PICUs is partially explained by these PICU-level factors. In addition, we will explore the potential interaction between trends in performance over time and the PICU-level covariates and staff insights. Given that we will enroll 10 PICUs in the study, we will limit the number of covariates included in each model and the interaction models should be considered exploratory in nature.

5.4 Interim Analyses

Interim analyses will be performed and presented to the DSMB every 12 months from the beginning of data collection. The interim analyses will focus on patient accrual and safety; data on trial operations, gender and minority inclusion and intervention effects will also be reviewed. The DSMB may recommend stopping the trial if: 1) The intervention is associated with increased safety events; 2) Patient accrual is well below acceptable goals and the ability of the study to achieve its goals is seriously compromised; or 3) evidence external to the study renders it unethical to continue the study. Given the minimal anticipated risk of the PICU Up intervention, no a priori stopping rules were specified for early stopping for safety or futility of the intervention.

6 Human Subjects

6.1 Institutional Review Board Reliance and Oversight

a. Single Institutional Review Board (sIRB) approval for the CCC and DCC and local IRB acknowledgment is required and will be secured before any subject is entered into the study at a given clinical site. IRB notices will be written and dated. The investigator will assure that s/he will promptly report to IRB all changes in the research activity and all unanticipated problems involving risk to human subjects or others, and that s/he will not make any changes in the research without IRB approval, except where necessary to eliminate apparent immediate hazard to human subjects (an event which is not expected to occur). Sites that are unable to participate in sIRB oversight will undergo oversight from their relevant IRB and will not enroll any patients until their local IRB approval has been received.

b. Johns Hopkins Medicine is serving as the single IRB for this study. It is the preference of Johns Hopkins Medicine IRB to use the SMART IRB reliance agreement as the basis of reliance. The SMART IRB master reliance agreement was created in 2016 to harmonize and streamline the IRB review process for multisite studies. It enables reliance on a study-by-study basis, clearly defines roles and responsibilities of relying institutions and reviewing IRBs, and eliminates the need to sign reliance agreements for each study [e.g., a non-SMART IRB agreement]. 900+ institutions have already signed onto this agreement and are actively using it as the basis of reliance for multisite projects. Sites that will rely on JHM IRB are still responsible for conducting a local context review prior to the start of research at their site and for following any local and institutionally required policies as it applies to research at their site (e.g., reporting of unanticipated problems).

6.2 Risks to Human Subjects

a. Human Subjects Involvement, Characteristics and Design

The proposed study is a stepped-wedge, cluster randomized trial of a pragmatic, interprofessional and multifaceted early mobility intervention (PICU Up!) conducted in 10 hospitals. The trial’s primary outcome is mechanical ventilation duration (through Day 21). Secondary outcomes include proportion of days with delirium and functional status at pediatric intensive care unit (PICU) discharge. Over a 2-year period, data will be collected on 1,440 PICU patients. The study includes an embedded process evaluation to identify factors associated with reliable PICU Up! delivery.

The PICU Up! trial will include 10 pediatric intensive care units (PICUs) in the United States, with each PICU acting as one cluster. In this traditional stepped-wedge design, all clusters begin in the control group and then transition to the intervention group at sequential and randomly assigned periods, facilitating the delivery of a desired intervention to all clusters, in this case, all participating PICUs. Thus, randomization will occur at the level of the PICU hospital unit. All participating PICUs will simultaneously start the baseline control period and data collection and plan their PICU Up! implementation process.

b. Study Procedures

PICU Up! is a multifaceted, interprofessional pathway that is integrated into routine PICU practice to safely optimize early and progressive patient mobility. PICU Up! incorporates the screening process for determining a patient's appropriate activity level into the daily rounding workflow for all eligible PICU patients, with a tiered activity plan based on clinical parameters to individualize goals based on each child's unique needs. While the patient's PICU Up! level is based on objective criteria, the interprofessional team collectively determines the daily activity goal(s) through shared decision-making, which is documented in the medical record on morning rounds. The intervention facilitates daily discussion of 1) goal-directed analgesia and sedation; 3) extubation readiness testing; 4) delirium screening and management; 5) sleep promotion; 6) family engagement and 7) activity goal including rehabilitation team consultation by PICU Day 3. As a pragmatic trial, all other aspects of PICU care will be conducted as per routine practice/at the medical team's discretion.

As a stepped-wedge cluster RCT and unit-based intervention, all patients admitted to the PICUs randomized to implementation, regardless of their length of stay, are screened for eligibility to receive the PICU Up! intervention, which includes criteria for exclusion based on specific clinical factors. Criteria for inclusion in data collection and outcomes evaluation for this trial will be children who are receiving mechanical ventilation via an endotracheal tube for ≥ 48 hours as of 7 am on Day 3 in the PICU. Exclusion criteria for data collection and outcomes evaluation are 1) active or anticipated withdrawal of life support within 48 hours; 2) open chest or abdomen; or 3) extracorporeal membrane oxygenation. Outcomes evaluation will be conducted for 720 children in the control/baseline period and 720 patients in the intervention (post-implementation) period.

Prior to and during the baseline data collection phase, the clinical sites will develop their PICU Up! implementation strategy with guidance and resources from the Clinical Coordinating Center (CCC). The sites will each develop an interprofessional PICU Up! champion team to lead the implementation in their unit. The implementation planning will focus on local barriers and facilitators to early and progressive mobility for critically ill patients as well as goal-directed sedation, delirium prevention, extubation readiness assessments, sleep promotion and family engagement. The PICU Up! Implementation Bundle includes discipline-specific educational resources (webinars, templates, educational presentations) and a unit-based electronic learning module to orient all PICU staff to PICU Up!.

Source of Materials: Sources of research material will include: 1) electronic health records; 2) organizational surveys completed by PICU leaders; and 3) document analysis.

Electronic health record: Trained research staff will review eligible patients' electronic health record daily to record the demographic, clinical, safety and PICU Up! performance data as outlined in the Research Strategy.

i. **Online surveys**: Two online surveys will be disseminated to PICU staff at each clinical site. The PICU Organizational and Resource Availability Survey will be completed by the clinical site PI in collaboration with medical, nursing and reha-

bilitation team leadership twice; once at the beginning of the study and again at the completion of the intervention data collection period.

ii. **Document Analysis**: All clinical sites will provide their PICU Up! related documentation forms and policies at the beginning of the study and end of the trial data collection phase in Aim 1.

All subject data will be maintained with strict privacy measures. Online surveys will be entered directly into a REDCap database. All data will be secured for the purpose of confidentiality (see **Adequacy of Protection Against Risks**, **Section 6.3**) and these data will only be used for research purposes.

c. Potential Risks

The PICU Up! intervention has very low potential risk to the patient. As a unit-based intervention focused on the interprofessional team's shared decision-making and workflow, the PICU Up! intervention integrates key aspects of PICU practice that are clinically accepted standard of care. This multifaceted care pathway integrates goal-directed analgesia and sedation, delirium monitoring and management, extubation readiness testing, sleep promotion, family engagement and early evaluation by the rehabilitation team into daily PICU care. Each of these practices are considered safe components of PICU care. All mobility activities and goals are individualized and determined solely by the patient's medical team and not the PICU Up! intervention. Moreover, all patients in the PICU are screened for PICU Up! eligibility regardless of their inclusion in data collection for this trial. The exclusion criteria include patients with special considerations including active or anticipated withdrawal of life support w/in 48 hours, open chest, open abdomen, and extracorporeal membrane oxygenation.

6.3 Adequacy of Protection Against Risks

a. Informed Consent and Assent

All patients who meet the eligibility criteria at participating sites will have data collected under a waiver of consent as outlined in **Section 3.1.e**.

b. Protections Against Risk

i. **PICU Up! Intervention**: As outlined above, the PICU Up! intervention poses no more than minimal risk. All of the components of the PICU Up! intervention are safe, including goal-directed analgesia and sedation, delirium screening, screening for extubation readiness, sleep promotion, family engagement and rehabilitation team consultation. Risk is further minimized as all mobilization activities are determined solely by the interprofessional PICU team.

ii. **Data Security and Confidentiality**: To minimize risks to patient confidentiality, each patient included in data collection will be assigned a unique study identification number, and all patient identifiers will be separated from the data obtained in the chart review. All Case Report Forms (CRFs) will be coded with this unique

study ID and not include other patient identifiers (e.g. names, medical record numbers). Each site’s enrollment log, linking Study ID Number to patient identity, will remain in a locked file in a locked office, accessible to the site PI and local research staff only. REDCap, the database for this study, has well documented and tested features to maintain data confidentiality and HIPAA compliance. Users must have valid login credentials (authentication), database access privileges and specific permissions within the database (authorization). Each site will only be able to access data from their own site in REDCap. Authentication and authorization can only be granted and revoked by authorized system administrators within the DCC. Only authorized study personnel will be granted access to enter and view study data, after completing training and receiving a personal login and password. A waiver of HIPAA Privacy Authorization will be requested to enable identification and screening of potential participants via review of Electronic Medical Record data.

Protected Health Information will not be reported in any publications nor disclosed outside the research team at each site. Access to electronic and hard copy files will be restricted to study staff members and investigators directly involved with the studies. Investigators associated with the study will be assigned separate unique passwords that are protected and will be known only to the individual user. The data system will maintain a log of all users that access it. Physical security of the workstations/files will be maintained. Automated daily data back-up plan will occur as per the Johns Hopkins network administrator’s standard protocol. The staff will receive training on data entry into the REDCap system and on the importance of security procedures. All key personnel will be required to complete human subjects research compliance training prior to joining the study, which includes a history of the IRB system and emphasizes the moral imperatives to protect the rights and wellbeing of research subjects. This training also includes a review of the federal regulations governing IRB operations and incorporates educational case studies and discussions of the ethical principles underlying research involving human subjects. The training has a specific emphasis on the informed consent procedure.

Data exported by the DCC from REDCap (e.g. for interval data cleaning, final data analyses) will be de-identified. This includes shifting dates, i.e. adjusting dates to not indicate actual dates. The shifting maintains time intervals to be able to calculate values such as length of stay or duration of mechanical ventilation.

6.4 Protocol Deviations and Violations

The investigator will not implement any deviation from, or changes of the protocol without agreement by the sponsor and prior review and documented approval/favorable opinion from the IRB of an amendment, except where necessary to eliminate immediate hazard(s) to study subjects, or when the change(s) involves only logistical or administrative aspects of the trial (e.g., change in monitor(s), change of telephone number(s)). Any deviation from the approved protocol will be documented and explained by the investigator or a person designated by the investigator. The investigator may implement a deviation from, or a change of, the protocol to eliminate immediate hazard(s) to trial

subjects without prior IRB approval/favorable opinion. As soon as possible, the implemented deviation or change, the reasons for it, and if appropriate, the proposed protocol amendment will be submitted to the IRB. A Protocol Deviation Summary Sheet (RF4) will be kept to detail minor, approved departures from protocol and will be included in the yearly Continuing Review Application.

6.5 Potential Benefit of the Research to Participants and Others

The proposed study has potential benefit for all patients admitted to participating PICUs. PICU Up! is a unit-based intervention which is directed at optimizing interprofessional collaboration to integrate goal-directed sedation, extubation readiness assessments, sleep promotion, delirium prevention, early mobility and family engagement into the daily PICU workflow. As a unit-based intervention, each child in the PICU is screened for PICU Up! eligibility regardless of their acuity of illness or mechanical ventilation status, and receives interventions based on their physiologic status and shared decision-making by the interprofessional team. While the proposed research will only include data from patients receiving invasive mechanical ventilation, this stepped-wedge cluster randomized trial includes implementation of the PICU Up! intervention for the entire PICU at the time of randomization. Thus, most patients in the PICU are eligible for the PICU Up! intervention and have potential for benefit from the proposed research.

6.6 Importance of the Knowledge to be Gained

This proposed study also has benefit to society because it is aimed at finding methods to improve the outcomes of pediatric survivors of critical illness, many of whom experience persistent physical, psychological and neurocognitive impairments. This study will contribute to understanding whether a multifaceted strategy to optimize early mobility affects duration of mechanical ventilation, delirium incidence and functional outcomes. Consequently, this study will generate important new knowledge regarding ways to optimize short and long-term outcomes for critically ill children.

7 Safety Monitoring

The PICU Up! intervention has low potential risk to the patient. As a unit-based intervention focused on the interprofessional team’s shared decision-making and workflow, the PICU Up! intervention integrates key aspects of PICU practice that are clinically accepted standard of care. This multifaceted care pathway integrates goal-directed analgesia and sedation, delirium monitoring and management, extubation readiness testing, sleep promotion, family engagement and early evaluation by the rehabilitation team into daily PICU care. Each of these practices are considered safe components of PICU care. All mobility activities and goals are individualized and determined solely by the patient’s medical team and not the PICU Up! intervention. Moreover, all patients in the PICU are screened for PICU Up! eligibility regardless of their inclusion in data collection for this trial. The exclusion criteria include patients with special considerations including active or anticipated withdrawal of life support w/in 48 hours, open chest, open abdomen, and extracorporeal membrane oxygenation. Any risks will be further minimized as described the section entitled **Adequacy of Protection Against Risks** (**Section 6.3**).

1. This pragmatic clinical trial will prospectively monitor all of the following events of interest identified via documentation in the electronic health record: a) falls; b) unplanned extubation or invasive line/tube removal; c) reintubation; and d) cardiac arrest.
2. These events of interest will be reviewed for trends and a summary of these events will be included be provided in reports to the DSMB meetings.
3. Relying sites should comply with their local reporting requirements as well as the Johns Hopkins reporting requirements, which can be found here:
   a. https://www.hopkinsmedicine.org/institutional_review_board/guidelines_policies/organization_policies/prompt_reporting_policy.html
   b. https://www.hopkinsmedicine.org/institutional_review_board/guidelines_policies/organization_policies/103_6a.html
4. An Event Report Summary Sheet (RF3) will be kept up to date and included in the yearly Continuing Review Application to summarize anticipated problems or events
5. Accepted definitions of Adverse Events (AEs) and Serious Adverse Events (SAEs) will be used as defined below:
   a. Adverse Event: Any untoward or unfavorable medical occurrence, including any abnormal sign, symptom or disease, whether or not considered related to the subject’s participation in the research.
   b. Serious Adverse Event- An adverse event will be considered “serious” if, in the view of the investigator, it results in any of the following outcomes: death, a life-threatening adverse reaction, prolongation of existing hospitalization, a persistent or significant incapacity, or substantial disruption of the ability to

conduct normal life functions. Important medical events that may not result in death or be life threatening may be considered serious when, based upon appropriate medical judgment, they may jeopardize the patient or subject and may require medical or surgical intervention to prevent one of the outcomes above.

6. We will report all safety events that fulfill criteria for a suspected, unexpected, serious adverse reaction (SUSAR) to the DSMB, IRB and NICHD. SUSARs are adverse events that are deemed by the site investigator to be both unexpected in nature and related to the patient's participation in the study. All SUSARs will be reported by the participating site to the DCC using the REDCap electronic data capture system. The DCC will generate an expedited SUSAR event report that will be submitted to the DSMB and other study stakeholders. Deidentified reporting will occur within 7 calendar days for such events that are fatal, life-threatening or serious (as per above definition) and within 10 calendar days for those which suggest greater risk of harm to participant than previously known or recognized.

7. Confidentiality of patients' data will be protected by having all patient-identifying information removed from databases before analysis. Electronic data storage will be password protected with daily back-up and storage. Only local research staff at each study site will have access to patient identifying information. All filing cabinets and storage facilities that contain sensitive patient information will be locked when not in use and all computers and storage cabinets will be located within secure office locations.

8 Publication Of Research Findings

Publication of the results of this trial will be governed by the policies and procedures developed by the Steering Committee. Any presentation, abstract, or manuscript will be made available for review by the sponsor and all study team investigators prior to submission.

9 References

1. Wieczorek B, Ascenzi J, Kim Y, Lenker H, Potter C, Shata NJ, et al. PICU Up!: Impact of a Quality Improvement Intervention to Promote Early Mobilization in Critically Ill Children. Pediatr Crit Care Med. 2016;17(12):e559-e66.

2. Randolph AG, Gonzales CA, Cortellini L, Yeh TS. Growth of pediatric intensive care units in the United States from 1995 to 2001. J Pediatr. 2004;144(6):792-8.

3. Pollack MM, Holubkov R, Reeder R, Dean JM, Meert KL, Berg RA, et al. PICU Length of Stay: Factors Associated With Bed Utilization and Development of a Benchmarking Model. Pediatr Crit Care Med. 2018;19(3):196-203.

4. Horak RV, Griffin JF, Brown AM, Nett ST, Christie LM, Forbes ML, et al. Growth and Changing Characteristics of Pediatric Intensive Care 2001-2016. Crit Care Med. 2019;47(8):1135-42.

5. Pollack MM, Holubkov R, Funai T, Clark A, Berger JT, Meert K, et al. Pediatric intensive care outcomes: development of new morbidities during pediatric critical care. Pediatr Crit Care Med. 2014;15(9):821-7.

6. Burns JP, Sellers DE, Meyer EC, Lewis-Newby M, Truog RD. Epidemiology of death in the PICU at five U.S. teaching hospitals*. Crit Care Med. 2014;42(9):2101-8.

7. Herrup EA, Wieczorek B, Kudchadkar SR. Characteristics of postintensive care syndrome in survivors of pediatric critical illness: A systematic review. World J Crit Care Med 2017;6(2):124-34.

8. Manning JC, Pinto NP, Rennick JE, Colville G, Curley MAQ. Conceptualizing Post Intensive Care Syndrome in Children-The PICS-p Framework. Pediatr Crit Care Med. 2018;19(4):298-300.

9. Choong K, Fraser D, Al-Harbi S, Borham A, Cameron J, Cameron S, et al. Functional Recovery in Critically Ill Children, the ”WeeCover” Multicenter Study. Pediatr Crit Care Med. 2018;19(2):145-54.

10. Anand KJ, Willson DF, Berger J, Harrison R, Meert KL, Zimmerman J, et al. Tolerance and withdrawal from prolonged opioid use in critically ill children. Pediatrics. 2010;125(5):e1208-25.

11. LaRosa JM, Aponte-Patel L. Iatrogenic Withdrawal Syndrome: a Review of Pathophysiology, Prevention, and Treatment. Current Pediatrics Reports. 2019;7(1):12-9.

12. Traube C, Silver G, Reeder RW, Doyle H, Hegel E, Wolfe HA, et al. Delirium in Critically Ill Children: An International Point Prevalence Study. Crit Care Med. 2017;45(4):584-90.

13. Faustino EVS, Shabanova V, Pinto MG, Li S, Trakas E, Miksa M, et al. Epidemiology of Lower Extremity Deep Venous Thrombosis in Critically Ill Adolescents. J Pediatr. 2018;201:176-83 e2.

14. Faustino EV, Raffini LJ. Prevention of Hospital-Acquired Venous Thromboembolism in Children: A Review of Published Guidelines. Front Pediatr. 2017;5:9.

15. Curley MA, Quigley SM, Lin M. Pressure ulcers in pediatric intensive care: incidence and associated factors. Pediatr Crit Care Med. 2003;4(3):284-90.

16. Cummins KA, Watters R, Leming-Lee T. Reducing Pressure Injuries in the Pediatric Intensive Care Unit. Nurs Clin North Am. 2019;54(1):127-40.

17. Field-Ridley A, Dharmar M, Steinhorn D, McDonald C, Marcin JP. ICU-Acquired Weakness Is Associated With Differences in Clinical Outcomes in Critically Ill Children. Pediatr Crit Care Med. 2016;17(1):53-7.

18. Kukreti V, Shamim M, Khilnani P. Intensive care unit acquired weakness in children: Critical illness polyneuropathy and myopathy. Indian J Crit Care Med. 2014;18(2):95-101.

19. Dervan LA, Di Gennaro JL, Farris RWD, Watson RS. Delirium in a Tertiary PICU: Risk Factors and Outcomes. Pediatr Crit Care Med. 2020;21(1):21-32.

20. Heneghan JA, Reeder RW, Dean JM, Meert KL, Berg RA, Carcillo J, et al. Characteristics and Outcomes of Critical Illness in Children With Feeding and Respiratory Technology Dependence. Pediatr Crit Care Med. 2019;20(5):417-25.

21. Elliott D, Davidson JE, Harvey MA, Bemis-Dougherty A, Hopkins RO, Iwashyna TJ, et al. Exploring the scope of post-intensive care syndrome therapy and care: engagement of non-critical care providers and survivors in a second stakeholders meeting. Crit Care Med. 2014;42(12):2518-26.

22. Harvey MA, Davidson J. Long-term consequences of critical illness: a new opportunity for high-impact critical care nurses. Crit Care Nurse. 2011;31(5):12-5.

23. Needham DM, Davidson J, Cohen H, Hopkins RO, Weinert C, Wunsch H, et al. Improving long-term outcomes after discharge from intensive care unit: report from a stakeholders' conference. Crit Care Med. 2012;40(2):502-9.

24. Fan E, Cheek F, Chlan L, Gosselink R, Hart N, Herridge MS, et al. An official American Thoracic Society Clinical Practice guideline: the diagnosis of intensive care unit-acquired weakness in adults. Am J Respir Crit Care Med. 2014;190(12):1437-46.

25. Haines KJ, Sevin CM, Hibbert E, Boehm LM, Aparanji K, Bakhru RN, et al. Key mechanisms by which post-ICU activities can improve in-ICU care: results of the international THRIVE collaboratives. Intensive care medicine. 2019;45(7):939-47.

26. Schweickert WD, Pohlman MC, Pohlman AS, Nigos C, Pawlik AJ, Esbrook CL, et al. Early physical and occupational therapy in mechanically ventilated, critically ill patients: a randomised controlled trial. Lancet. 2009;373(9678):1874-82.

27. Burtin C, Clerckx B, Robbeets C, Ferdinande P, Langer D, Troosters T, et al. Early exercise in critically ill patients enhances short-term functional recovery. Crit Care Med. 2009;37(9):2499-505.

28. Kayambu G, Boots R, Paratz J. Physical therapy for the critically ill in the ICU: a systematic review and meta-analysis. Crit Care Med. 2013;41(6):1543-54.

29. Needham DM, Korupolu R, Zanni JM, Pradhan P, Colantuoni E, Palmer JB, et al. Early physical medicine and rehabilitation for patients with acute respiratory failure: a quality improvement project. Arch Phys Med Rehabil. 2010;91(4):536-42.

30. Morris PE, Goad A, Thompson C, Taylor K, Harry B, Passmore L, et al. Early intensive care unit mobility therapy in the treatment of acute respiratory failure. Crit Care Med. 2008;36(8):2238-43.

31. Bailey P, Thomsen GE, Spuhler VJ, Blair R, Jewkes J, Bezdjian L, et al. Early activity is feasible and safe in respiratory failure patients. Crit Care Med. 2007;35(1):139-45.

32. Damluji A, Zanni JM, Mantheiy E, Colantuoni E, Kho ME, Needham DM. Safety and feasibility of femoral catheters during physical rehabilitation in the intensive care unit. J Crit Care. 2013;28(4):535 e9-15.

33. Kho ME, Damluji A, Zanni JM, Needham DM. Feasibility and observed safety of interactive video games for physical rehabilitation in the intensive care unit: a case series. J Crit Care. 2012;27(2):219 e1-6.

34. Kho ME, Truong AD, Zanni JM, Ciesla ND, Brower RG, Palmer JB, et al. Neuromuscular electrical stimulation in mechanically ventilated patients: a randomized, sham-controlled pilot trial with blinded outcome assessment. J Crit Care. 2015;30(1):32-9.

35. Sricharoenchai T, Parker AM, Zanni JM, Nelliot A, Dinglas VD, Needham DM. Safety of physical therapy interventions in critically ill patients: a single-center prospective evaluation of 1110 intensive care unit admissions. J Crit Care. 2014;29(3):395-400.

36. Korupolu R, Gifford JM, Zanni JM, Truong AD, Vajrala G, Needham DM. Early mobilization in the intensive care unit: safety, feasibility and benefits. In: Esquinas AM, editor. Applied Technologies in Pulmonary Medicine. Basel: Karger; 2011.

37. Denehy L, Skinner EH, Edbrooke L, Haines K, Warrillow S, Hawthorne G, et al. Exercise rehabilitation for patients with critical illness: a randomized controlled trial with 12 months of follow-up. Crit Care. 2013;17(4):R156.

38. Preiser J-C, De Prato C, Harvengt A, Peters L, Bastin M-H, Fraipont V, et al. Passive Cycling Limits Myofibrillar Protein Catabolism in Unconscious Patients: A Pilot Study. J Nov Physiother. 2014;4(4):1-6.

39. Morris PE, Berry MJ, Files DC, Thompson JC, Hauser J, Flores L, et al. Standardized rehabilitation and hospital length of stay among patients with acute respiratory failure. Jama. 2016;315(24):2694-702.

40. Schujmann DS, Teixeira Gomes T, Lunardi AC, Zoccoler Lamano M, Fragoso A, Pimentel M, et al. Impact of a Progressive Mobility Program on the Functional Status, Respiratory and Muscular Systems of ICU Patients: A Randomized and Controlled Trial. Crit Care Med. 2019.

41. Kudchadkar S, Nelliot A, Awojoodu R, Vaidya D, Traube C, Walker T, et al. Physical Rehabilitation in Critically Ill Children: A Multicenter Point Prevalence Study in the United States. Crit Care Med. 2020:In press.

42. Choong K, Foster G, Fraser DD, Hutchison JS, Joffe AR, Jouvet PA, et al. Acute rehabilitation practices in critically ill children: a multicenter study. Pediatr Crit Care Med. 2014;15(6):e270-9.

43. Miura S, Wieczorek B, Lenker H, Kudchadkar SR. Normal Baseline Function Is Associated With Delayed Rehabilitation in Critically Ill Children. J Intensive Care Med. 2018:885066618754507.

44. Cui LR, LaPorte M, Civitello M, Stanger M, Orringer M, Casey F, 3rd, et al. Physical and occupational therapy utilization in a pediatric intensive care unit. J Crit Care. 2017;40:15-20.

45. Hopkins RO, Choong K, Zebuhr CA, Kudchadkar SR. Transforming PICU Culture to Facilitate Early Rehabilitation. J Pediatr Intensive Care. 2015;4(4):204-11.

46. Donovan AL, Aldrich JM, Gross AK, Barchas DM, Thornton KC, Schell-Chaple HM, et al. Interprofessional Care and Teamwork in the ICU. Crit Care Med. 2018;46(6):980-90.

47. Zheng K, Sarti A, Boles S, Cameron S, Carlisi R, Clark H, et al. Impressions of Early Mobilization of Critically Ill Children-Clinician, Patient, and Family Perspectives. Pediatr Crit Care Med. 2018;19(7):e350-e7.

48. October TW, Hinds PS, Wang J, Dizon ZB, Cheng YI, Roter DL. Parent Satisfaction With Communication Is Associated With Physician's Patient-Centered Communication Patterns During Family Conferences. Pediatr Crit Care Med. 2016;17(6):490-7.

49. Levin AB, Fisher KR, Cato KD, Zurca AD, October TW. An Evaluation of Family-Centered Rounds in the PICU: Room for Improvement Suggested by Families and Providers. Pediatr Crit Care Med. 2015;16(9):801-7.

50. White DB, Angus DC, Shields AM, Buddadhumaruk P, Pidro C, Paner C, et al. A Randomized Trial of a Family-Support Intervention in Intensive Care Units. N Engl J Med. 2018;378(25):2365-75.

51. Slonim AD, Pollack MM. Integrating the Institute of Medicine's six quality aims into pediatric critical care: relevance and applications. Pediatr Crit Care Med. 2005;6(3):264-9.

52. Dhurjati R, Sigurdson K, Profit J. Patient- and Family-Centered Care as a Dimension of Quality. Am J Med Qual. 2019;34(3):307-8.

53. Vasilevskis EE, Ely EW, Speroff T, Pun BT, Boehm L, Dittus RS. Reducing iatrogenic risks: ICU-acquired delirium and weakness–crossing the quality chasm. Chest. 2010;138(5):1224-33.

54. Kudchadkar SR, Yaster M, Punjabi NM. Sedation, Sleep Promotion, and Delirium Screening Practices in the Care of Mechanically Ventilated Children: A Wake-Up Call for the Pediatric Critical Care Community*. Critical Care Medicine. 2014;42(7):1592-600.

55. Kudchadkar SR, Aljohani OA, Punjabi NM. Sleep of critically ill children in the pediatric intensive care unit: a systematic review. Sleep Med Rev. 2014;18(2):103-10.

56. Traube C, Silver G, Gerber LM, Kaur S, Mauer EA, Kerson A, et al. Delirium and Mortality in Critically Ill Children: Epidemiology and Outcomes of Pediatric Delirium. Crit Care Med. 2017;45(5):891-8.

57. Curley MA, Wypij D, Watson RS, Grant MJ, Asaro LA, Cheifetz IM, et al. Protocolized sedation vs usual care in pediatric patients mechanically ventilated for acute respiratory failure: a randomized clinical trial. JAMA. 2015;313(4):379-89.

58. Saelim K, Chavananon S, Ruangnapa K, Prasertsan P, Anuntaseree W. Effectiveness of Protocolized Sedation Utilizing the COMFORT-B Scale in Mechanically Ventilated Children in a Pediatric Intensive Care Unit. J Pediatr Intensive Care. 2019;8(3):156-63.

59. Dreyfus L, Javouhey E, Denis A, Touzet S, Bordet F. Implementation and evaluation of a paediatric nurse-driven sedation protocol in a paediatric intensive care unit. Ann Intensive Care. 2017;7(1):36.

60. Gaillard-Le Roux B, Liet JM, Bourgoin P, Legrand A, Roze JC, Joram N. Implementation of a Nurse-Driven Sedation Protocol in a PICU Decreases Daily Doses of Midazolam. Pediatr Crit Care Med. 2017;18(1):e9-e17.

61. Awojoodu R, Kudchadkar S, Injury P-PIAL, Investigators S. 824: FACTORS ASSOCIATED WITH FAMILY PRESENCE AT THE BEDSIDE OF CRITICALLY ILL INFANTS AND CHILDREN. Critical Care Medicine. 2020;48(1):392.

62. Kudchadkar SR, Aljohani O, Johns J, Leroux A, Alsafi E, Jastaniah E, et al. Day-Night Activity in Hospitalized Children after Major Surgery: An Analysis of 2271 Hospital Days. J Pediatr. 2019;209:190-7 e1.

63. Kudchadkar SR, Yaster M, Punjabi AN, Quan SF, Goodwin JL, Easley RB, et al. Temporal Characteristics of the Sleep EEG Power Spectrum in Critically Ill Children. J Clin Sleep Med. 2015;11(12):1449-54.

64. Flannery AH, Oyler DR, Weinhouse GL. The Impact of Interventions to Improve Sleep on Delirium in the ICU: A Systematic Review and Research Framework. Crit Care Med. 2016;44(12):2231-40.

65. Ely EW. The ABCDEF Bundle: Science and Philosophy of How ICU Liberation Serves Patients and Families. Crit Care Med. 2017;45(2):321-30.

66. Pun BT, Balas MC, Barnes-Daly MA, Thompson JL, Aldrich JM, Barr J, et al. Caring for Critically Ill Patients with the ABCDEF Bundle: Results of the ICU Liberation Collaborative in Over 15,000 Adults. Crit Care Med. 2019;47(1):3-14.

67. Barnes-Daly MA, Phillips G, Ely EW. Improving Hospital Survival and Reducing Brain Dysfunction at Seven California Community Hospitals: Implementing PAD Guidelines Via the ABCDEF Bundle in 6,064 Patients. Crit Care Med. 2017;45(2):171-8.

68. Balas MC, Burke WJ, Gannon D, Cohen MZ, Colburn L, Bevil C, et al. Implementing the awakening and breathing coordination, delirium monitoring/management, and early exercise/mobility bundle into everyday care: opportunities, challenges, and lessons learned for implementing the ICU Pain, Agitation, and Delirium Guidelines. Crit Care Med. 2013;41(9 Suppl 1):S116-27.

69. Campbell MK, Piaggio G, Elbourne DR, Altman DG, Group C. Consort 2010 statement: extension to cluster randomised trials. BMJ. 2012;345:e5661.

70. Grayling MJ, Wason JM, Mander AP. Stepped wedge cluster randomized controlled trial designs: a review of reporting quality and design features. Trials. 2017;18(1):33.

71. Hemming K, Haines TP, Chilton PJ, Girling AJ, Lilford RJ. The stepped wedge cluster randomised trial: rationale, design, analysis, and reporting. BMJ. 2015;350:h391.

72. Hargreaves JR, Prost A, Fielding KL, Copas AJ. How important is randomisation in a stepped wedge trial? Trials. 2015;16:359-.

73. R Core Team. R: A Language and Environment for Statistical Computing. Vienna, Austria: R Foundation for Statistical Computing; 2014.

74. Fiser DH. Assessing the outcome of pediatric intensive care. J Pediatr. 1992;121(1):68-74.

75. Pollack MM, Holubkov R, Funai T, Dean JM, Berger JT, Wessel DL, et al. The Pediatric Risk of Mortality Score: Update 2015. Pediatr Crit Care Med. 2016;17(1):2-9.

76. Balas MC, Vasilevskis EE, Olsen KM, Schmid KK, Shostrom V, Cohen MZ, et al. Effectiveness and safety of the awakening and breathing coordination, delirium monitoring/management, and early exercise/mobility bundle. Crit Care Med. 2014;42(5):1024-36.

77. Curley MA, Harris SK, Fraser KA, Johnson RA, Arnold JH. State Behavioral Scale: a sedation assessment instrument for infants and young children supported on mechanical ventilation. Pediatr Crit Care Med. 2006;7(2):107-14.

78. Hoyer EH, Young DL, Klein LM, Kreif J, Shumock K, Hiser S, et al. Toward a Common Language for Measuring Patient Mobility in the Hospital: Reliability and Construct Validity of Interprofessional Mobility Measures. Phys Ther. 2018;98(2):133-42.

79. Traube C, Silver G, Kearney J, Patel A, Atkinson TM, Yoon MJ, et al. Cornell Assessment of Pediatric Delirium: a valid, rapid, observational tool for screening delirium in the PICU*. Crit Care Med. 2014;42(3):656-63.

80. Needham DM, Sinopoli DJ, Dinglas VD, Berenholtz SM, Korupolu R, Watson SR, et al. Improving data quality control in quality improvement projects. Int J Qual Health Care. 2009;21(2):145-50.

81. Harris PA, Taylor R, Thielke R, Payne J, Gonzalez N, Conde JG. Research electronic data capture (REDCap)–a metadata-driven methodology and workflow process for providing translational research informatics support. J Biomed Inform. 2009;42(2):377-81.

82. Smith HA, Boyd J, Fuchs DC, Melvin K, Berry P, Shintani A, et al. Diagnosing delirium in critically ill children: Validity and reliability of the Pediatric Confusion Assessment Method for the Intensive Care Unit. Crit Care Med. 2011;39(1):150-7.

83. McPherson MLM. Demystifying opioid conversion calculations : a guide for effective dosing. 2nd ed. Bethesda, MD: American Society of Health-System Pharmacists; 2018.

84. Barr J, Zomorodi K, Bertaccini EJ, Shafer SL, Geller E. A double-blind, randomized comparison of i.v. lorazepam versus midazolam for sedation of ICU patients via a pharmacologic model. Anesthesiology. 2001;95(2):286-98.

85. Holden RJ, Carayon P, Gurses AP, Hoonakker P, Hundt AS, Ozok AA, et al. SEIPS 2.0: a human factors framework for studying and improving the work of healthcare professionals and patients. Ergonomics. 2013;56(11):1669-86.

86. Health NIo. PICU Up!: Pilot Stepped-Wedge Trial of a Multicomponent Early Mobility Intervention for Critically Ill Children 2019 [Available from: https://clinicaltrials.gov/ct2/show/NC

87. Hussey MA, Hughes JP. Design and analysis of stepped wedge cluster randomized trials. Contemp Clin Trials. 2007;28(2):182-91.

88. Zhang Z. Multiple imputation with multivariate imputation by chained equation (MICE) package. Ann Transl Med. 2016;4(2):30.
